# Supplementary material for: LINC01413/hnRNP-K/ZEB1 Axis Accelerates Cell Proliferation and EMT in Colorectal Cancer via Inducing YAP1/TAZ1 Translocation
Source: Mol Ther Nucleic Acids. 2019 Nov 29;19:546–61. doi: 10.1016/j.omtn.2019.11.027 (PMC6953771; doi:10.1016/j.omtn.2019.11.027)

OMTN, Volume 19

Supplemental Information

LINC01413/hnRNP-K/ZEB1 Axis Accelerates Cell Proliferation and EMT in Colorectal Cancer via Inducing YAP1/TAZ1 Translocation

Ling Ji, Xiang Li, Zhenhua Zhou, Zhihai Zheng, Li Jin, and Feizhao Jiang

Supplement figure 1 Heat maps of differentially expressed mRNA. The differentially expressed mRNA profiles are analyzed using high throughput chip technology. Red means highly expressed and blue means low expressed. Besides, this is the enlarged image of Fig. 4A.

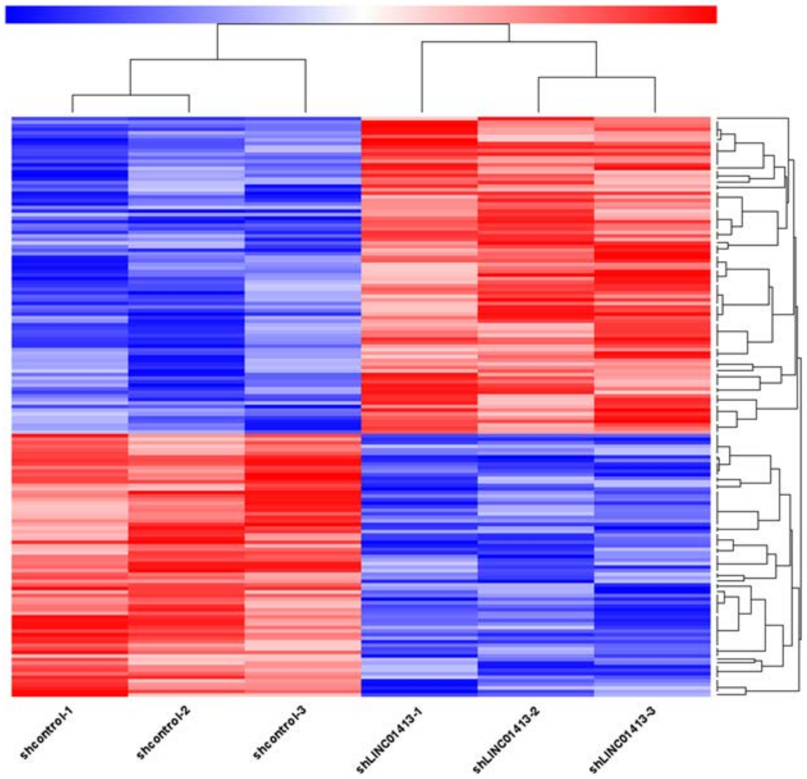

Supplement figure 2 LINC01413 mainly plays a role in Hippo pathway and cell migration process. The possible pathways or processes that could be regulated by LINC01413 which are determined by GO and pathway analysis. Besides, this is the enlarged image of Fig. 4B.

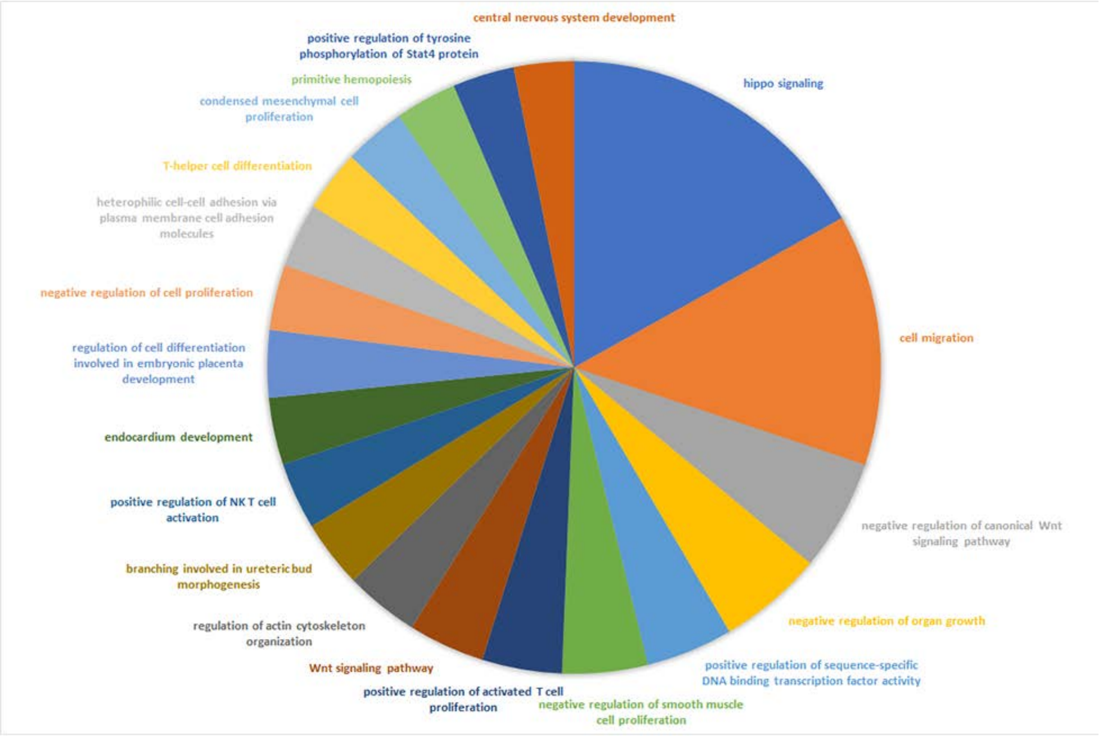

Supplement figure 3 Transcription of ZEB1 is suggested to be regulated by TEAD4. (A) UCSC predicated TEAD4 as a potential transcription factor for ZEB1. (B) The binding motif of TEAD4 obtained from JASPAR.

A

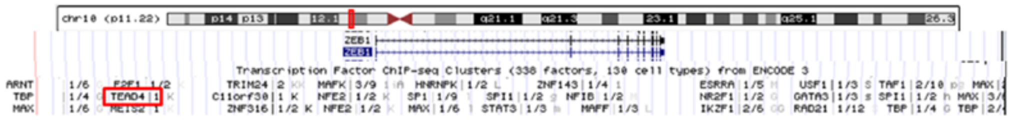

B

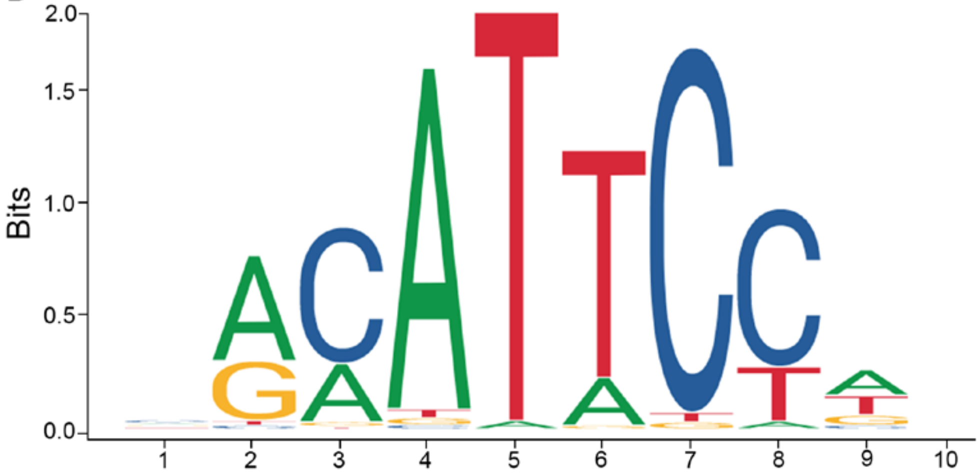

Supplement figure 4 LINC01413 regulates ZEB1 expression through hnRNP-K/YAP1/TAZ1 axis. (A-B) The impact of LINC01413 on two well-known targets of YAP/TAZ is estimated by luciferase reporter assay (A) and ChIP assay (B). (C) The result of qRT–PCR reveal that knockdown or overexpression of hnRNP-K has no influence on LINC01413 expression. (D) Western blot assay is utilized to study the effect of hnRNP-K silence on the protein levels of ZEB1, YAP1 and TAZ1 as well as the distribution of YAP1 and TAZ1 in LoVo cells. (E) Western blot results show that overexpression of hnRNP-K in HT-29 cells promotes the level of ZEB1, p-YAP1 and p-TAZ1 as well as the translocation of YAP1/TAZ1. (F) RNA expression of LINC01413, ZEB1, YAP1 and TAZ1 in tumors obtained from in vivo experiments is tested by qRT-PCR, and the protein levels of ZEB1, YAP1, TAZ1, p-YAP1 (Ser^{127}) and p-TAZ1 (Ser^{89}) in above tumors are detected through western blot. Error bars show the mean ±SD of more than three independent experiments. **$P < 0.01$ vs. control group.

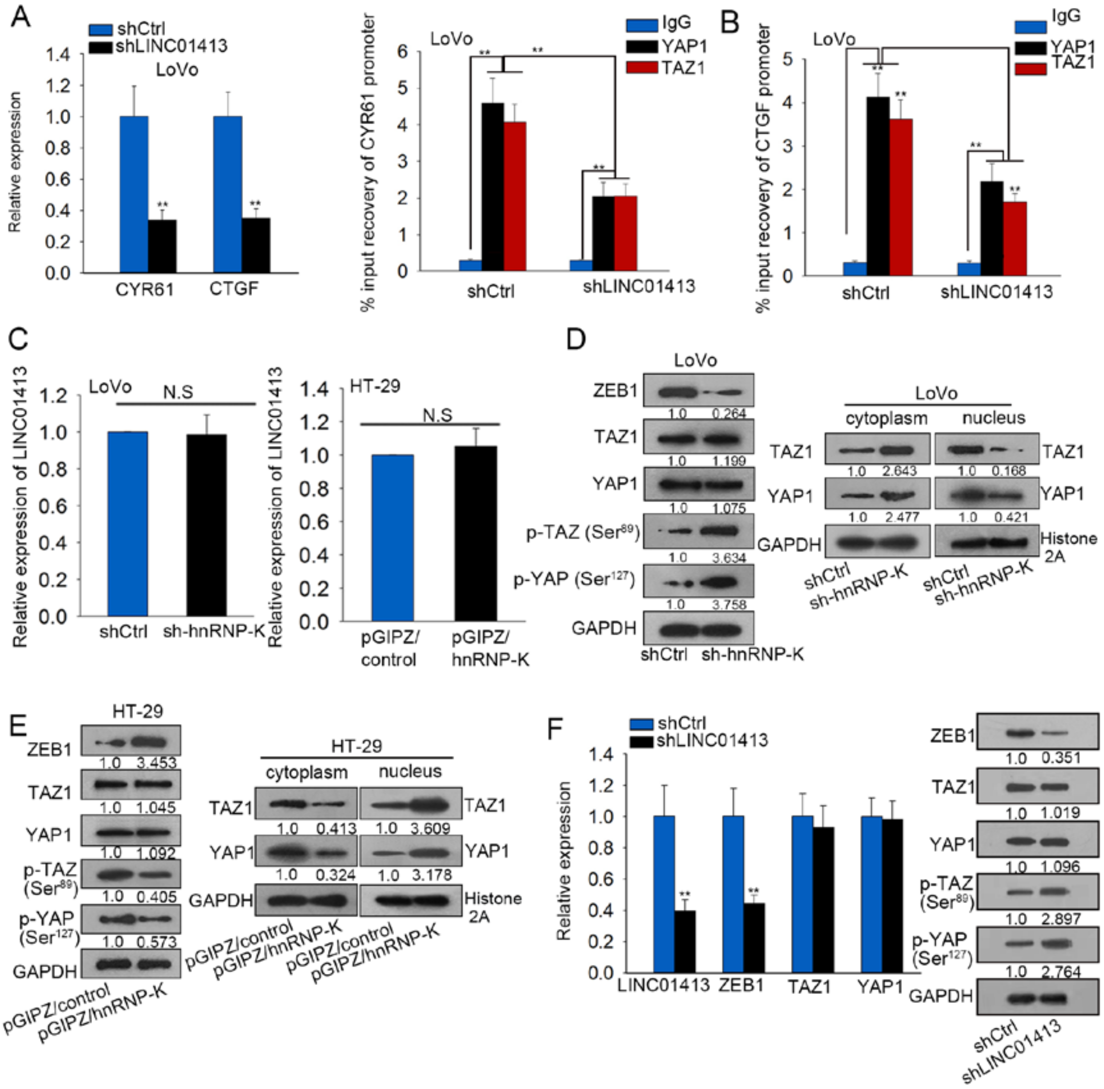
A
shCtrl
shLINC01413
LoVo
Relative expression
CYR61
CTGF
% input recovery of CYR61 promoter
IgG
YAP1
TAZ1
shCtrl
shLINC01413
B
LoVo
% input recovery of CTGF promoter
IgG
YAP1
TAZ1
shCtrl
shLINC01413
C
LoVo
N.S
Relative expression of LINC01413
shCtrl
sh-hnRNP-K
HT-29
N.S
pGIPZ/
control
pGIPZ/
hnRNP-K
D
LoVo
ZEB1
TAZ1
YAP1
p-TAZ (Ser89)
p-YAP (Ser127)
GAPDH
shCtrl
sh-hnRNP-K
LoVo
cytoplasm
nucleus
TAZ1
YAP1
GAPDH
Histone
2A
shCtrl
sh-hnRNP-K
E
HT-29
ZEB1
TAZ1
YAP1
p-TAZ
(Ser89)
p-YAP
(Ser127)
GAPDH
pGIPZ/control
pGIPZ/hnRNP-K
HT-29
cytoplasm
nucleus
TAZ1
YAP1
GAPDH
Histone
2A
pGIPZ/control
pGIPZ/hnRNP-K
F
shCtrl
shLINC01413
Relative expression
LINC01413
ZEB1
TAZ1
YAP1
ZEB1
TAZ1
YAP1
p-TAZ
(Ser89)
p-YAP
(Ser127)
GAPDH
shCtrl
shLINC01413

Supplementary Figure 5 Quantification of protein bands in Figure 2L (A), Figure 3F (B), Figure 4C (C), Figure 4D (D), Figure 5H (E), Figure 6A (F), Figure 6B (G), Figure 6G (H), Figure S4D (I), Figure S4E (J), Figure S4F (K). **P < 0.01 vs. control group.

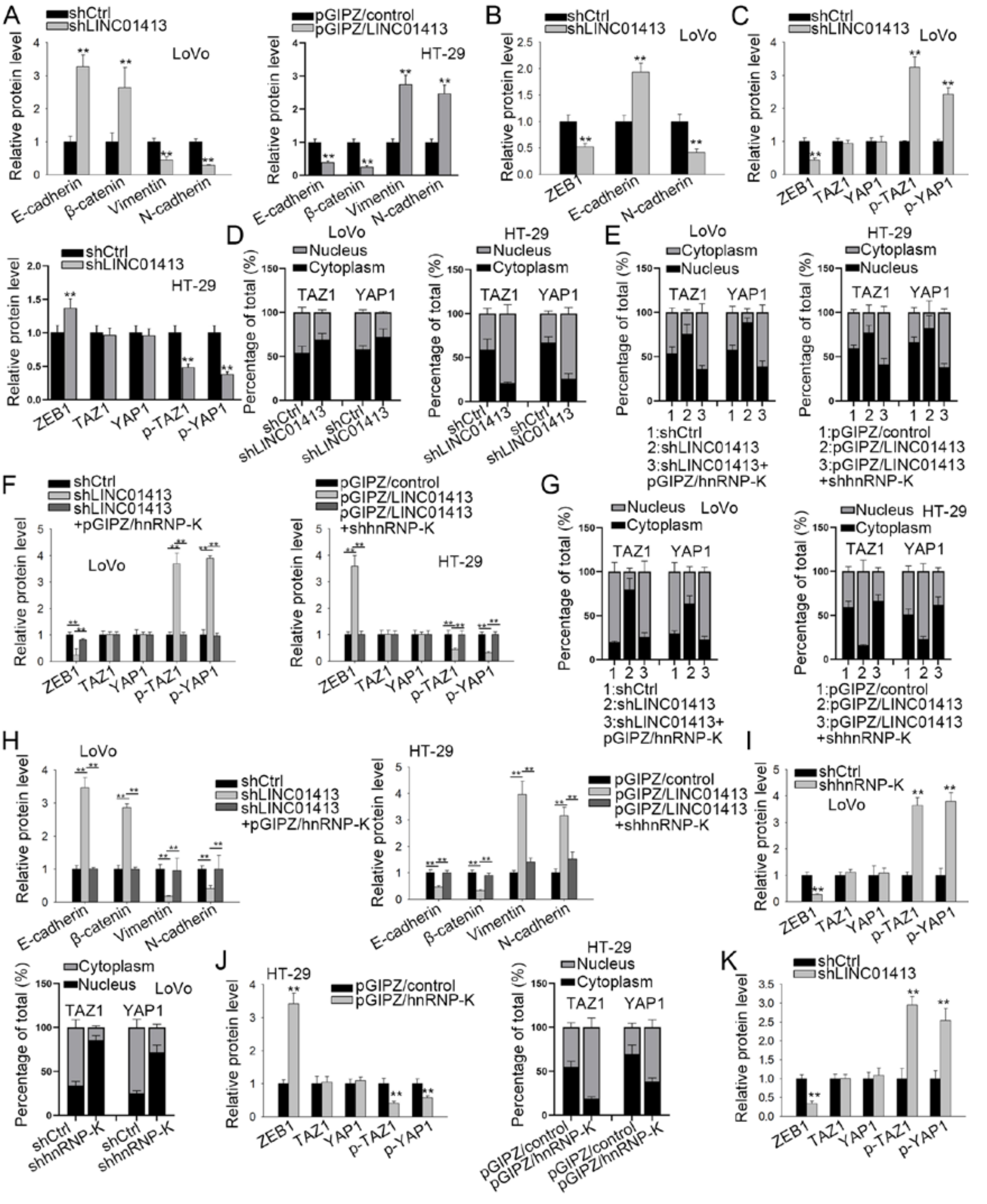

Supplement: Document S1. Figures S1–S5 [file mmc1.pdf]
