# Supplementary material for: LINC01413/hnRNP-K/ZEB1 Axis Accelerates Cell Proliferation and EMT in Colorectal Cancer via Inducing YAP1/TAZ1 Translocation
Source: Mol Ther Nucleic Acids. 2019 Nov 29;19:546–61. doi: 10.1016/j.omtn.2019.11.027 (PMC6953771; doi:10.1016/j.omtn.2019.11.027)

Molecular Therapy
Nucleic Acids
Original Article

AMERICAN SOCIETY of GENE & CELL THERAPY

LINC01413/hnRNP-K/ZEB1 Axis Accelerates Cell Proliferation and EMT in Colorectal Cancer via Inducing YAP1/TAZ1 Translocation

Ling Ji,[1] Xiang Li,[2] Zhenhua Zhou,[1] Zhihai Zheng,[1] Li Jin,[1] and Feizhao Jiang[1]

[1]The First Hospital of Wenzhou Medical University, Wenzhou, Zhejiang 325035, China; [2]Zhejiang Provincial Key Laboratory of Medical Genetics, Wenzhou Medical University, Wenzhou, Zhejiang 325035, China

Long non-coding RNAs (lncRNAs) are crucial molecules in tumorigenesis and tumor growth in various human cancers, including colorectal cancer (CRC). Studies have revealed that lncRNAs can regulate cellular processes in cancers by interacting with proteins, for example RNA-binding proteins (RBPs). In this study, we recognize a novel lncRNA called LINC01413 that is upregulated in CRC tissues through lncRNAs microarray. Subsequently, we confirmed that an elevated level of LINC01413 expression in CRC tissues was strongly correlated to clinicopathological features, such as tumor size, tumor stage, lymph node metastasis, and distant metastasis, and its association with poor overall survival was also revealed. Additionally, LINC01413 facilitates cell proliferation, migration, invasion, and epithelial-mesenchymal transition (EMT) *in vitro*. Also, silenced LINC01413 restrains tumor growth *in vivo*. Moreover, LINC01413 binds with hnRNP-K and induces YAP1 (yes-associated protein 1)/TAZ1 (tafazzin) nuclear translocation to regulate the expression of ZEB1 in CRC cells. Taken together, this research suggested LINC01413 as a positive regulator in CRC progression through the LINC01413/hnRNP-K/TAZ1/YAP1/ZEB1 axis, broadening a new view on CRC treatment.

INTRODUCTION

Colorectal cancer (CRC) is the third most prevalent and lethal type of malignancy in the world.[1] Despite great improvements in diagnostic and therapeutic techniques, the prognosis of CRC patients remains poor.[2–4] Actually, about 90% of mortality from CRC is due to distant metastasis, and epithelial-mesenchymal transition (EMT) is noted as the major contributing factor of metastasis.[5]

Long non-coding RNAs (lncRNAs) are transcripts with more than 200 nt that have no protein-coding potential.[6] Increasing evidence has illustrated that lncRNAs are involved in many diseases and that dysregulation of lncRNAs contributes to tumorigenesis in various cancers.[7–9] Recently, lncRNAs have been reported as an EMT modulator in cancers. For instance, lncRNA plays important roles in the EMT process in ovarian cancer.[10] lncRNA XLOC_000647 exerts functions in EMT-induced cell invasion via downregulating NLRP3.[11] In addition, several lncRNAs have also been proven to influence EMT in CRC. As an example, TUG1 triggers EMT in CRC by prompting KIAA1199 expression via miR-600.[12] Also, LINC01133 restrains EMT and metastasis in CRC by interacting with SRSF6.[13] LINC01413 is a novel lncRNA that locates in 15q21.3, and its role or function in CRC remains unclear.

The EMT, once thought to be essential during embryonic development, has been increasingly reported in human cancers recently due to its role in tumor invasion and metastasis.[14,15] The full process of EMT involves a complex genetic program, including losing epithelial character and acquiring mesenchymal and motility properties.[5] Normal or tumor epithelial cells initiate EMT once separated from their neighbors and migrating away.[16,17] There are several factors that have been regarded as main regulators of EMT, such as Snail, ZEB, and Twist families.[17–19] Additionally, the expression of these regulators is stringently modulated by various cell-intrinsic pathways and extracellular signals in the processes of transcription and translation, as well as protein stability control.[20,21] Moreover, recent studies have certified that lncRNAs or microRNAs (miRNAs) could also regulate the occurrence of EMT.[22,23] For example, lincRNA-ROR is an EMT inducer and a contributor to the tumorigenesis and metastasis of breast cancer.[24] However, the regulation network of EMT is still not well understood, and thus it requires further study.

The Hippo pathway is evidenced as typical anti-tumor signaling during cancer development.[25] Activation of this pathway is determined by the phosphorylation of two core factors, YAP1 (yes associated protein 1) and TAZ1 (tafazzin).[26] YAP and TAZ are critical transcriptional regulators that are pervasively activated and essential for the initiation and development of human malignancies.[27] Normally, YAP and TAZ are phosphorylated by LATS1 or LATS2 so that they are unable to translocate into nucleus, leading to transrepression of their targets, which play a role during tumorigenesis and cancer progression.[28] Furthermore, the Hippo pathway has also been revealed to exert important functions in intestinal disease, including CRC.[29]

Received 3 February 2019; accepted 18 November 2019;
https://doi.org/10.1016/j.omtn.2019.11.027.

Correspondence: Feizhao Jiang, The First Hospital of Wenzhou Medical University, Wenzhou, Zhejiang 325035, China.
E-mail: feizhaojiang_zfz@163.com

This is an open access article under the CC BY license (http://creativecommons.org/licenses/by/4.0/).

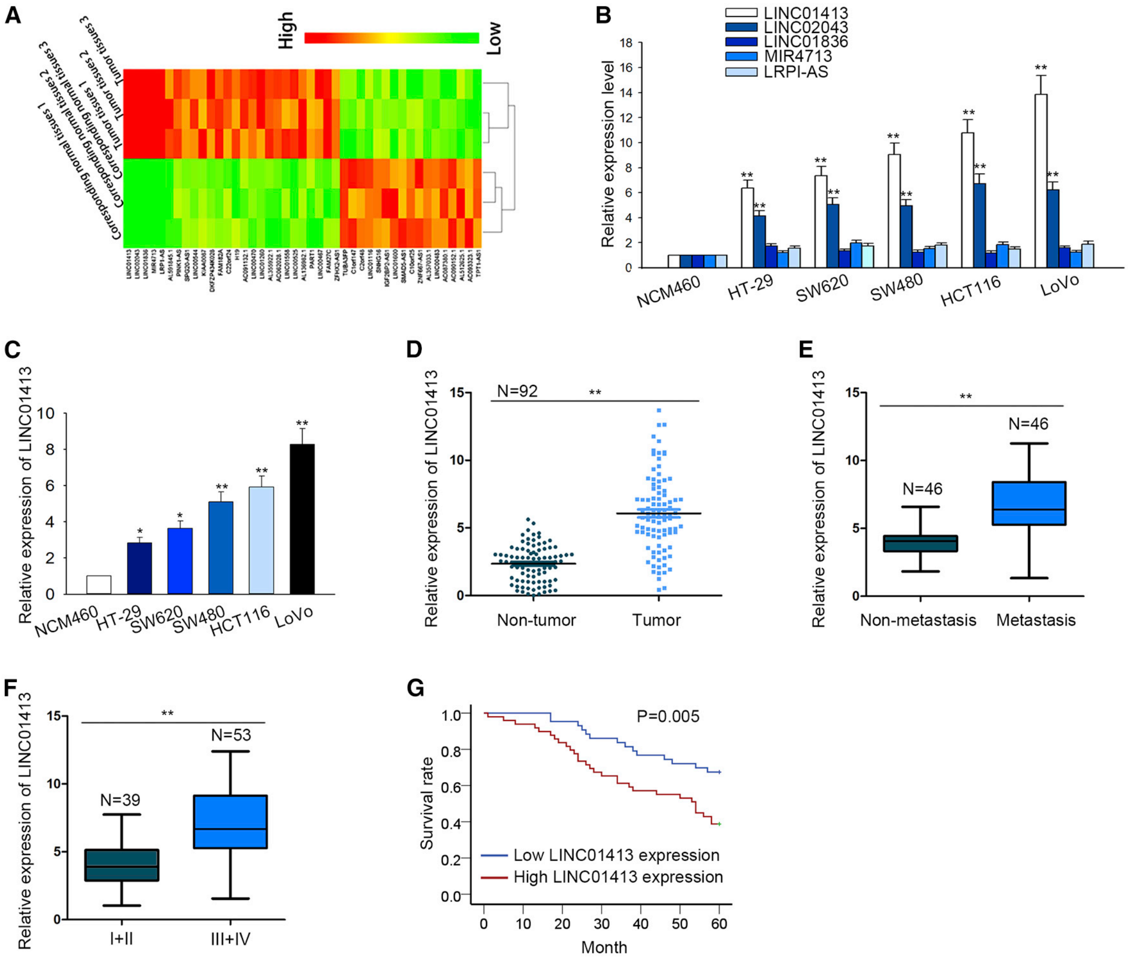

Figure 1. LINC01413 Is Upregulated in CRC Tissues and Cell Lines and Indicates Poor Prognosis for CRC Patients

(A) High-throughput RNA sequencing of total cellular RNA obtained from three pairs of CRC tissues and corresponding normal tissues was performed to explore the transcriptome changes in CRC. (B) Quantitative real-time PCR results of the expression of the five most altered lncRNAs in CRC cell lines. (C) Relative high level of LINC01413 was determined in CRC cells compared with normal NCM460 cell. Result was obtained using quantitative real-time PCR. (D) Quantitative real-time PCR detected the higher LINC01413 expression in CRC tissues than that in non-tumor tissues. (E) The expression levels of LINC01413 in tissues with or without distant metastasis were accessed by quantitative real-time PCR. (F) LINC01413 expression in different TNM stages of CRC was evaluated by quantitative real-time PCR. (G) Kaplan-Meier analysis (log-rank test) was used to estimate the relationship between LINC01413 expression and overall survival of CRC patients. Error bars show the mean ± SD of more than three independent experiments. $^{*}p < 0.05$, $^{**}p < 0.01$ versus control group.

In this study, we investigated the specific role as well as the potential mechanism of LINC01413 in CRC.

RESULTS

LINC01413 Is Upregulated in CRC Tissues and Cell Lines and Indicates Poor Prognosis for CRC Patients

To explore the transcriptome changes in CRC, total RNAs obtained from three pairs of CRC tissues and corresponding normal tissues were analyzed by using high-throughput RNA sequencing. We revealed that a total of 473 lncRNAs are differentially expressed, among which 43 exhibit more than 15-fold variation, including 26 lncRNAs with high expression and 17 lncRNAs with low expression (Figure 1A). Then, we tested the five most upregulated lncRNAs among the 26 above-mentioned candidates in CRC cell lines, including LINC01413, LINC02043, LINC01836, MIR4713, and LRP1-AS. As a result, LINC01413 and LINC02043 were found to be highly expressed while the other three genes changed little in five CRC cell lines compared to the normal colon epithelial cell line NCM460 (Figure 1B). Based on these results, we further explored the role of LINC01413 in CRC, which exhibited the highest expression in CRC cell lines among the five lncRNAs detected.

First, we proved again that LINC01413 expression is notably enhanced in CRC cell lines (Figure 1C). Furthermore, we also observed LINC01413 upregulation in 92 of the CRC tissues in comparison with paired non-tumor tissues (Figure 1D). In addition,

Table 1. Correlation between LINC01413 Expression and Clinical Features of CRC Patients

	LINC01413 Expression		
Variable	Low	High	p Value
**Age (Years)**			
6	34	31	0.113
≥60	9	18	
**Sex**			
Male	14	14	0.821
Female	29	35	
**Tumor Size (cm)**			
≤5	28	15	0.002
5	15	34	
**TNM Stage**			
I–II	25	14	0.006
III–IV	18	35	
**Lymph Node Metastasis**			
Negative	27	19	
Positive	16	30	0.036
**Distant Metastasis**			
Negative	23	14	0.019
Positive	20	35	

Low/high by the sample mean. Pearson χ^2 test. $p < 0.05$ was considered statistically significant. n = 92 patients.

Table 2. Multivariate Analysis of Prognostic Parameters in Patients with CRC by Cox Regression Analysis

Variable	Category	p Value
Age (years)		
	<60	0.498
	≥60	
Sex		
	male	0.333
	female	
Tumor size (cm)		
	≤5	0.197
	>5	
TNM stage		
	I–II	0.032
	III–IV	
Lymph node metastasis		
	negative	0.019
	positive	
Distant metastasis		
	negative	0.775
	positive	
LINC01413 level	low	0.034
	high	

Proportional hazards method analysis showed a positive, independent prognostic importance of LINC01413 expression (p = 0.034). $p < 0.05$ was considered statistically significant.

CRC tissues with distant metastasis showed higher levels of LINC01413 expression than did those without distant metastasis (Figure 1E). Moreover, LINC01413 level seemed to be closely related to TNM (tumor, node, metastasis) stages because patients at a more advanced stage usually expressed a high LINC01413 level in contrast to those at earlier stages (Figure 1F). These data suggest that LINC01413 may function as an oncogene in CRC.

To further analyze the role of LINC01413 in CRC, the 92 CRC patients with multiple clinicopathological characteristics were divided into two groups according to high (n = 49) or low (n = 43) LINC01413 expression. As shown in Table 1, high levels of LINC01413 were accompanied with large tumor size (p = 0.002), higher TNM stage (p = 0.006), lymph node metastasis (p = 0.036), and distant metastasis (p = 0.019) in patients, whereas its expression hardly correlated with age or sex. Moreover, the Cox proportional hazards model applied in multivariate analysis elucidated that LINC01413 expression (p = 0.034) can serve as an independent predictor for CRC prognosis, as can TNM stage (p = 0.032) and lymph node metastasis (p = 0.019) (Table 2). Furthermore, the results of Kaplan-Meier analysis and a log-rank test implied that higher LINC01413 expression tended to lead to poorer overall survival (Figure 1G). These observations suggested that enhanced LINC01413 may play a role in the progression of CRC.

LINC01413 Regulates Cell Proliferation, Apoptosis, Migration, Invasion, and EMT in CRC Cells

To make sure of the role of LINC01413 in CRC progression, a short hairpin RNA (shRNA) targeting LINC01413 was designed and transfected into LoVo cells, and the cells exhibited a relatively low level of LINC01413 (Figure 2A, left). Meanwhile, LINC01413 was overexpressed in HT29 cells after transfecting with pGIPZ/LINC01413 (Figure 2A, right). The 3-(4,5-dimethylthiazol-2-yl)-2,5-dimethyltetrazolium bromide (MTT) assay results indicated that silencing LINC01413 remarkably inhibited cell viability compared with the negative control shRNA group in LoVo cells, whereas overexpression of LINC01413 in HT29 cells exhibited an opposite effect (Figure 2B). In comparison with the negative controls, knockdown of LINC01413 increased the proportion of apoptotic cells, whereas overexpression of LINC01413 apparently hampered cell apoptosis (Figures 2C and 2D). In other words, LINC01413 promotes CRC cell growth *in vitro*.

To explore the effect of LINC01413 on tumor metastasis in CRC, a Transwell assay was first employed *in vitro* to test cell migration and invasion capacities. Compared with the negative control, cell migratory ability was evidently suppressed after silencing

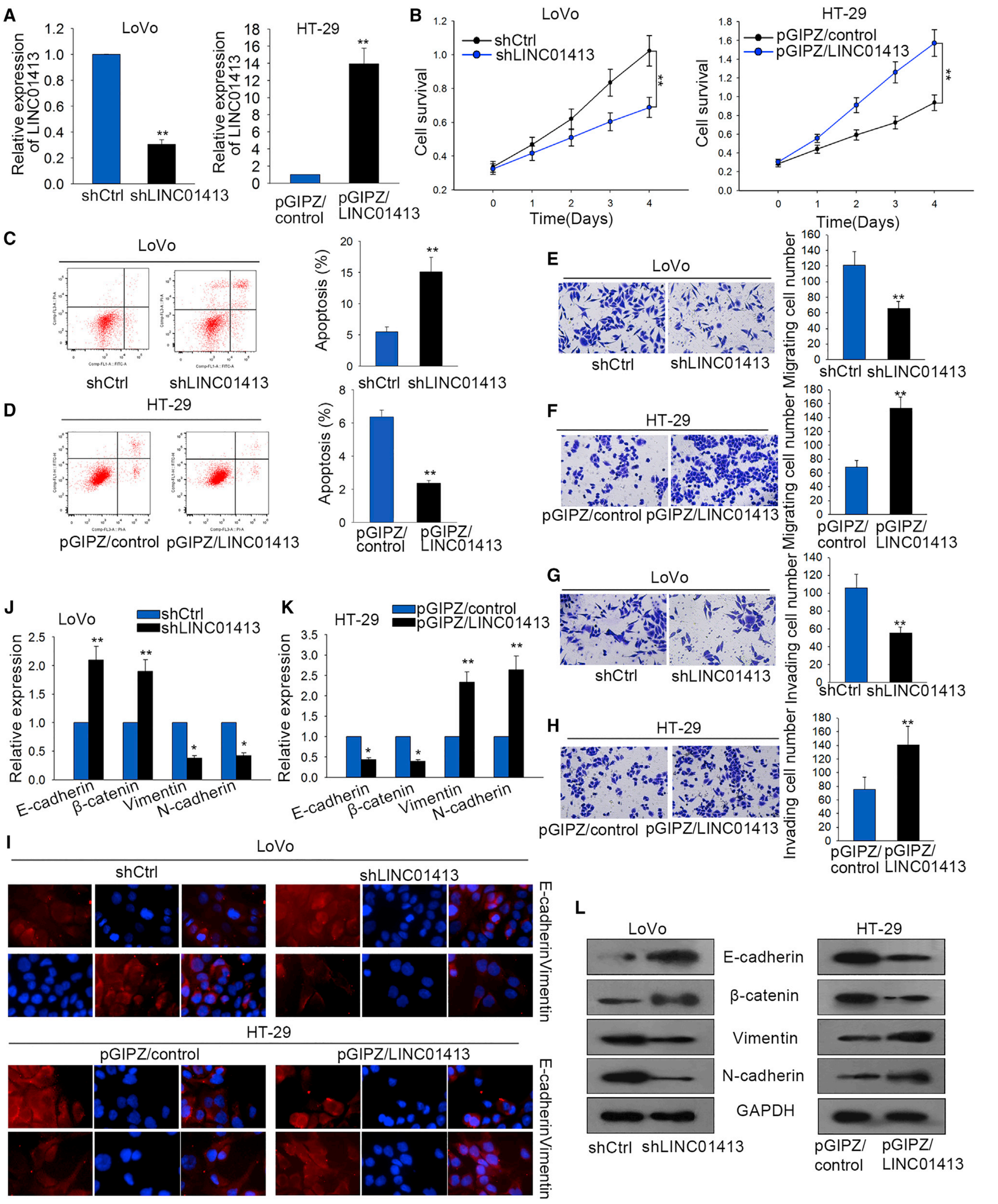

(legend on next page)

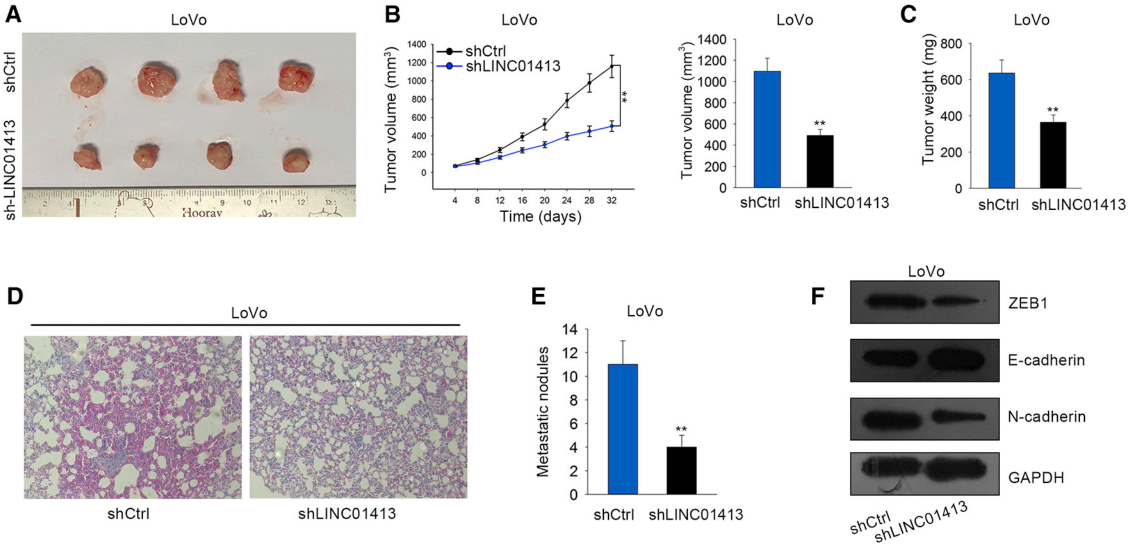

Figure 3. LINC01413 Regulates Tumorigenesis and Tumor Metastasis of CRC *In Vivo*

(A) Images of tumors formed from shCtrl or shLINC01413-transfected LoVo cells. (B and C) shLINC01413 transfection decreased the volume tumor (B) and weight (C) *in vivo*. (D-E) Number of metastasis nodules in shLINC01413 group was more than shCtrl group. (F) Western blot reveals that the level of E-cadherin is remarkably increased while the level of ZEB1 and N-cadherin is decreased in tumors originating from LINC01413-silenced LoVo cells when compared to tumors from shCtrl-transfected cells. Error bars show the mean ± SD of more than three independent experiments. **p < 0.01 versus control group.

LINC01413 in LoVo cells, and, conversely, it was markedly enhanced in LINC01413-overexpressed HT29 cells (Figures 2E and 2F). Similarly, the invasion ability of CRC cells was controlled along with LINC01413 knockdown while being strengthened along with overexpression, just as for the same trend of cell migratory ability (Figures 2G and 2H). Furthermore, we assessed whether LINC01413 influences EMT, a hallmark of metastasis in CRC. The immunofluorescence (IF) staining showed that LINC01413 inhibition increased the expression of E-cadherin but decreased that of Vimentin, while LINC01413 upregulation diminished Vimentin expression but improved E-cadherin level (Figure 2I). Furthermore, silencing LINC01413 caused an increased expression of epithelial markers, including E-cadherin and β-catenin, whereas it decreased the levels of mesenchymal markers such as Vimentin and N-cadherin in LoVo cells; however, overexpression of LINC01413 showed an inverse impact on the expression of these genes (Figures 2J and 2K). Consistently, the results of western blots further justified the observations above (Figure 2L). These investigations reveal that LINC01413 contributes to tumor metastasis of CRC.

LINC01413 Knockdown Blocks Tumorigenesis and Tumor Metastasis of CRC *In Vivo*

To further validate the promoting role of LINC01413 in CRC cell growth and metastasis, LINC01413-silenced LoVo cells were injected into nude mice while the shCtrl-transfected LoVo cells acted as the control. Consequently, the *in vivo* xenograft experiments demonstrated that tumors originating from shLINC01413-transfected cells are remarkably smaller than those from control cells (Figure 3A). As shown in Figures 3B and 3C, obvious reductions of tumor volume and weight were found in tumors derived from cells with LINC01413 silencing compared to those from controls. Moreover, the *in vivo* metastasis assays represented that LINC01413 inhibition markedly lessened the metastatic tumors secondary to the lung when compared to the lungs of control mice (Figures 3D and 3E). Moreover, silenced LINC01413 significantly increased E-cadherin expression but decreased N-cadherin expression, and the level of ZEB1 protein, which plays a key part in EMT, was also confined under LINC01413 depletion (Figure 3F). Altogether, we illustrated that LINC01413 serves an oncogenic and metastasis-promoting role *in vivo*.

Figure 2. LINC01413 Regulates Cell Proliferation, Apoptosis, Migration, Invasion, and EMT in CRC Cells

(A) LoVo cells transfected with shLINC01413 show a relatively low level of LINC01413 (left), and HT-29 cells transfected with pGIPZ/LINC01413 exhibit a relatively high expression of LINC01413 (right). (B) MTT assay revealed that knockdown of LINC01413 inhibits cell survival, whereas overexpression of LINC01413 enhances cell survival, compared to the control groups. (C and D) Flow cytometry analysis was used to determine the apoptosis rate of LoVo (C) or HT-29 (D) cells after transfection. (E-H) Transwell assays demonstrated that cells with higher level of LINC01413 with the higher migratory and invasive ability compared with cells with lower level of LINC01413. (I) Immunofluorescence staining indicates that the dysregulation of LINC01413 affects the distribution of E-cadherin and N-cadherin in CRC cells. (J and K) The expression levels of EMT-associated genes in transfected CRC cells were detected by quantitative real-time PCR. (L) Western blot assays were performed to detect the protein levels of the epithelial and mesenchymal markers in response to LINC01413 inhibition or overexpression. Error bars show the mean ± SD of more than three independent experiments. *p < 0.05, **p < 0.01 versus control group.

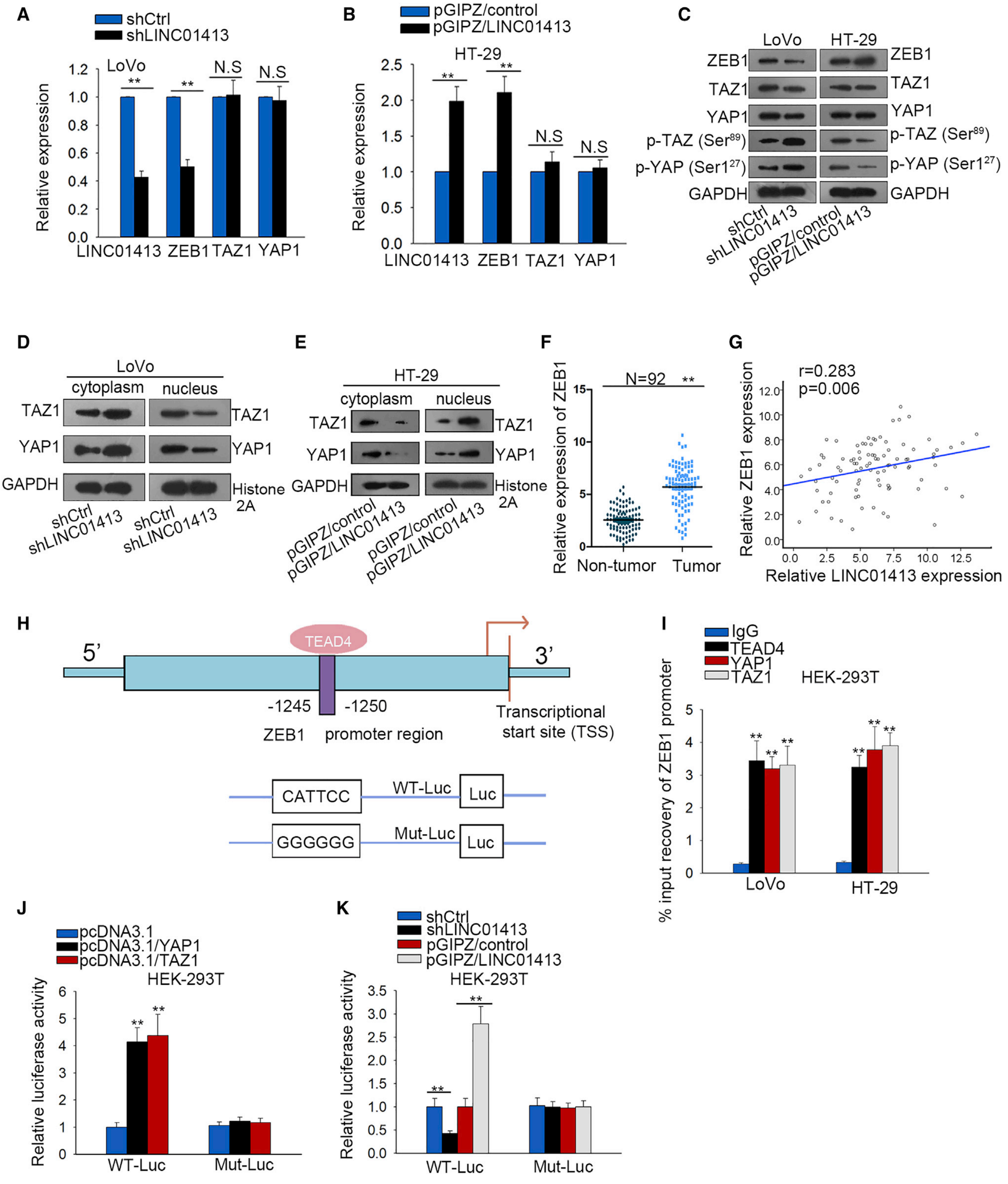

(legend on next page)

LINC01413 Modulates ZEB1 Expression through Promoting YAP1/TAZ1 Complex Translocation into Nucleus

To probe the underlying pathway involved in the function of LINC01413 on CRC progression, we first screened the mRNA differential expression profiles of LoVo and HCT-116 cell lines before and after LINC01413 interference by high-throughput microarray technology. It was found that a total of 673 mRNAs were dysregulated (fold change $\geq$2.0), among which 214 were upregulated and 459 were downregulated (Figure S1). Then, the results from Gene Ontology (GO) and pathway analysis revealed that LINC01413 function is mainly related to the Hippo signaling pathway and cell migration (Figure S2). Then, the Pearson correlation coefficient between LINC01413 and other differentially encoded genes was calculated and we found that LINC01413 was significantly correlated with ZEB1, which can initiate EMT. Therefore, we hypothesized a regulatory relationship between LINC01413, ZEB1, and the Hippo signaling pathway during formation of the malignant phenotype in CRC.

To validate this hypothesis, we first examined whether LINC01413 regulates the expression of ZEB1, YAP1, and TAZ1 by quantitative real-time PCR and western blot analysis. As shown in Figure 4A, ZEB1 expression declined significantly, whereas the expression of YAP1 and TAZ1 barely changed with the knockdown of LINC01413 in LoVo cells. However, overexpression of LINC01413 upregulated ZEB1, whereas it failed to alter the expression of YAP1 and TAZ1 (Figure 4B). Next, the western blot results showed that the protein levels of ZEB1, YAP1, and TAZ1 changed in the same trend as mRNA levels, except that the phosphorylation levels of YAP1 (at site Ser^{127}) and TAZ1 (at site Ser^{89}) were greatly upregulated in LINC01413-silenced LoVo cells but downregulated in response to LINC01413 overexpression (Figure 4C). These findings implied that LINC01413 hinders YAP/TAZ phosphorylation so as to inactivate Hippo pathway.

As is well known, the YAP/TAZ complex has been revealed to exert its carcinogenic function in cancers through the transcriptional activation of target genes in the nucleus. Therefore, we detected the level of YAP1 and TAZ1 in the cytoplasm and nucleus using western blot assay. As a consequence, YAP1 and TAZ1 were found to be highly expressed in cytoplasm and expressed at low levels in nucleus in LINC01413-silenced LoVo cells, whereas they were expressed at low levels in cytoplasm and highly expressed in nucleus in LINC01413-overexpressed HT-29 cells, which indicated that LINC01413 affects the intranuclear and extranuclear distribution of YAP1 and TAZ1 (Figures 4D and 4E). Furthermore, we also discovered that ZEB1, an EMT activator that has been reported as a target of YAP/TAZ,[30] was remarkably upregulated in CRC tissues and was positively correlated with LINC01413 in 92 CRC tissues (Figures 4F and 4G). Moreover, the UCSC genome browser suggested that TEAD4 is a possible transcription factor regulating ZEB1 transcription (Figure S3A). According to the TEAD4-binding motif obtained from JASPAR (http://jaspar.genereg.net/) (Figure S3B), we discovered that TEAD4 is most likely to bind to the ZEB1 promoter at the −1245 to −1250 site upstream of the transcriptional start site (TSS) (Figure 4H). Furthermore, we observed that the ZEB1 promoter is highly enriched by anti-TEAD4, anti-YAP1, and anti-TAZ1 (Figure 4I). Also, we suggest that the luciferase activity of the ZEB1 promoter was clearly enhanced under YAP1 and TAZ1 overexpression or LINC01413 upregulation, but it was reduced under LINC01413 inhibition (Figures 4J and 4K), proving that LINC01413 reinforces ZEB1 transcription via a YAP/TAZ-mediated manner. Additionally, it seems that LINC01413 also influences the transcription of CYR61 and CTGF, two well-known targets downstream YAP/TAZ, because we observed that LINC01413 depletion decreased the binding of YAP/TAZ to their promoters and consequently resulted in the decline of luciferase activities of their promoters (Figures S4A and S4B). These findings suggest that LINC01413 upregulates ZEB1 expression in CRC cells through enhancing the transcriptional activity of the YAP1/TAZ1 complex.

LINC01413 Stimulates Nuclear Translocation of the YAP1/TAZ1 Complex by Binding with hnRNP-K

Recently, many reports have revealed that lncRNAs can regulate gene expression through interacting with proteins. Thus, we suspected that LINC01413 may have an impact on YAP/TAZ in such a way. Through an RNA pull-down assay and mass spectrometry, we found that there are 27 proteins interacting with LINC01413, among which hnRNP-K showed the highest binding score (Figure 5A; Table 3), and the interactivity of LINC01413 with hnRNP-K was further confirmed by RNA pull-down and an RNA immunoprecipitation (RIP) assay (Figures 5B and 5C). Through prediction by the online tool RNA-Protein Interaction Prediction (RPISeq) (http://pridb.gdcb.iastate.edu/RPISeq/), we found that LINC01413 has a high potential to bind with hnRNP-K at 0–198 bp upstream of the 3′ end of LINC01413 (shown in red in Box 1), among which the sequence at 67–98 bp upstream of the 3′ end of LINC01413 (further highlighted in yellow in Box 1) maintains the highest conservatism and the highest binding potential (Box 1). More importantly, it exhibited that

Figure 4. LINC01413 Modulates ZEB1 Expression through Promoting YAP1/TAZ1 Complex Translocation into Nucleus

(A and B) The expressions of LINC01413, ZEB1, YAP1, and TAZ1 in LoVo cells transfected with shLINC01413 or shCtrl (A) and in HT-29 cells transfected with pGIPZ/LINC01413 or pGIPZ/control (B) were detected by quantitative real-time PCR. (C) The protein levels of ZEB1, YAP1, and TAZ1 in LoVo and HT-29 cells after transfection were estimated by western blot assay. (D and E) Western blot results of the expression of TAZ1 and YAP1 in cytoplasm or nucleus of LoVo (D) and HT-29 (E) cells after silencing or overexpressing LINC01413, respectively. (F) Quantitative real-time PCR analysis of ZEB1 expression in CRC tissues and adjacent non-tumor tissues. (G) Spearman's correlation analysis suggests a positive correlation between ZEB1 expression and LINC01413 in CRC tissues. (H) The binding site of TEAD4 in the region of ZEB1 promoter was predicted by JASPAR. (I) ChIP assay proves that TEAD4, YAP1, and TAZ1 all bind to the ZEB1 promoter. (J and K) Luciferase reporter assays were performed to evaluate the function of YAP1/TAZ1 (J) or LINC01413 (K) in ZEB1 transcription. Error bars show the mean ± SD of more than three independent experiments. **$p < 0.01$ versus control group.

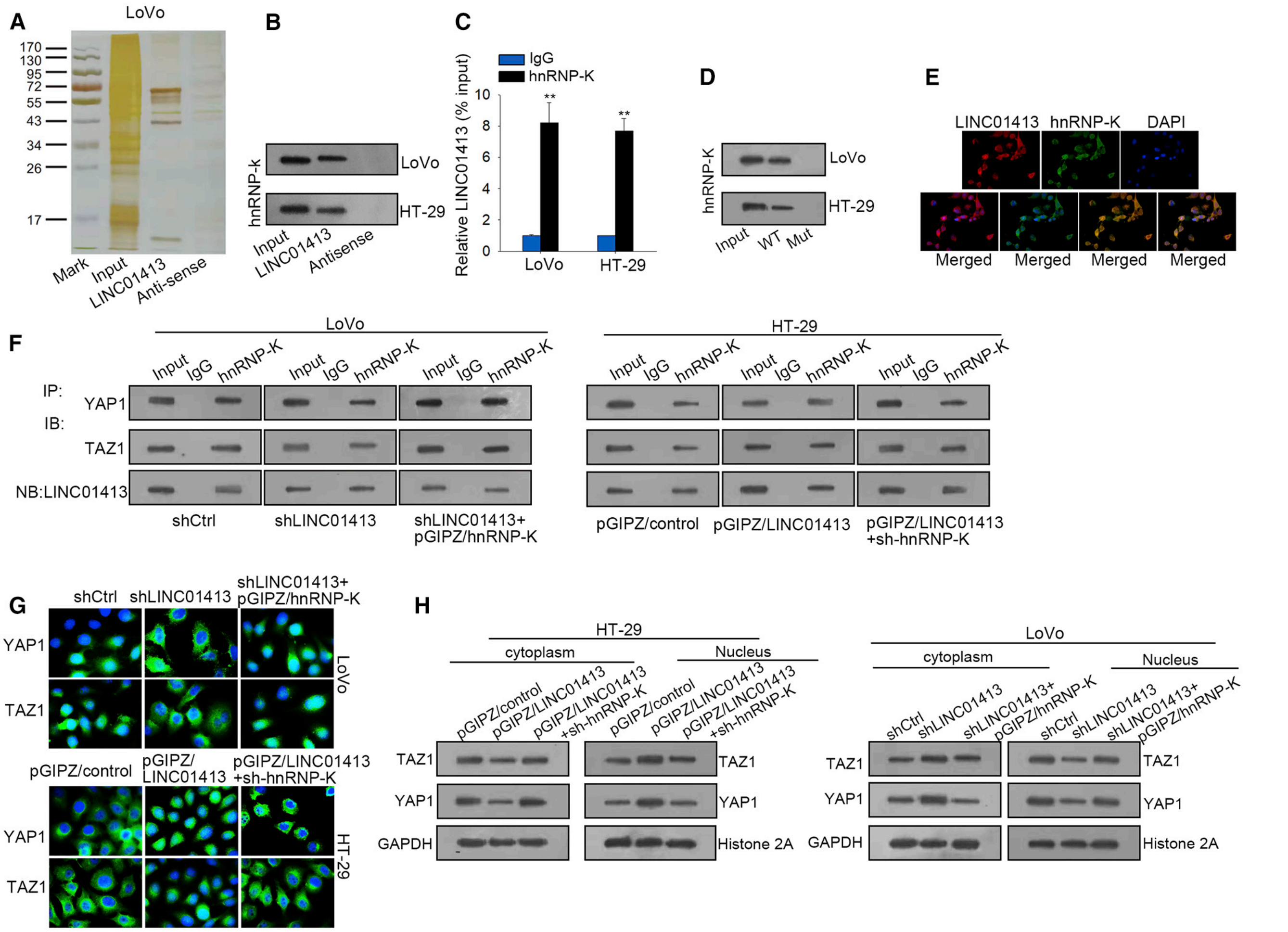

Figure 5. LINC01413 Stimulates ZEB1 Expression and the Nuclear Translocation of the YAP1/TAZ1 Complex by Binding with hnRNP-k

(A) A western blot assay after a RNA pull-down assay was applied to test protein interaction with LINC01413. (B) RNA pull-down showed the enrichment of hnRNP-k in response to LINC01413 compared with negative control IgG. (C) RIP assay detected the enrichment of LINC01413 in anti-hnRNP-k group compared with anti-IgG. (D) The specific interaction between LINC01413 and hnRNP-K was confirmed by an RNA pull-down assay. (E) The locations of LINC01413 and hnRNP-K in LoVo cells were assessed using FISH. (F) The cross-talk among LINC01413, hnRNP-K, YAP1, and TAZ1 in CRC cells was identified via a coIP assay. (G and H) The impact of the LINC01413/hnRNP-K axis on the nuclear translocation of YAP and TAZ was assessed by IF staining (G) and subcellular fractionation followed by western blot analysis (H). Error bars show the mean ± SD of more than three independent experiments. **p < 0.001 versus control group.

hnRNP-K no longer interacts with LINC01413 when the sequence 67–98 bp upstream of the 3′ end of LINC01413 mutated (Figure 5D). Moreover, the fluorescence *in situ* hybridization (FISH) results represented that both LINC01413 and hnRNP-K are expressed not only in the nucleus but also in the cytoplasm, and, more importantly, the co-localization of these two genes was also displayed here (Figure 5E). These data reveal the direct interaction between LINC01413 and hnRNP-K in CRC cells.

To explore whether LINC01413 exerts its effects on CRC via hnRNP-K, the following experiments were conducted. First, we found that altering hnRNP-K expression had no influence on LINC01413 expression (Figure S4C). Interestingly, silencing hnRNP-K led to noticeable downregulation of ZEB1, stimulated phosphorylation of YAP1 as well as TAZ1, and blocked their nuclear translocation, with total levels of YAP1 and TAZ1 rarely changed (Figure S4D), similar to results observed in LoVo cells upon LINC01413 silencing (Figure 4E). Nevertheless, opposite phenomena were shown in hnRNP-K-overexpressed HT-29 cells (Figure S4E). These results highlight that LINC01413 stimulates YAP1/TAZ1 nuclear translocation by relying on hnRNP-K to modulate YAP1/TAZ1/ZEB1 signaling.

Next, we sought to identify the detailed mechanism whereby the LINC01413/hnRNP-K axis affects YAP1/TAZ1 nuclear translocation. Due to the ability of hnRNP-K to interact with a range of molecular partners, including DNA, RNA, and proteins, we speculated that LINC01413 promotes YAP1/TAZ1 nuclear translocation

Table 3. The Potential Chaperonins for LINC01413

	Protein	Score	Mass	Matches	Sequences
1	hnRNP-K_HUMAN	998	224,394	181 (62)	106 (44)
2	Q8WZ42_HUMAN	861	3,842,904	261 (68)	244 (67)
3	P16615_HUMAN	321	116,336	43 (20)	33 (16)
4	P25705_HUMAN	307	59,828	37 (15)	24 (13)
5	P63261_HUMAN	232	42,108	32 (12)	16 (9)
6	P35609_HUMAN	217	104,358	42 (12)	33 (12)
7	P10809_HUMAN	200	61,187	12 (6)	12 (6)
8	Q00839_HUMAN	194	91,269	45 (14)	23 (11)
9	E9PKE3_HUMAN	171	68,991	24 (9)	17 (8)
10	A8MXZ9_HUMAN	171	141,774	18 (9)	14 (7)
11	Q12906_HUMAN	166	95,678	35 (12)	21 (9)
12	Q08211_HUMAN	161	142,181	33 (14)	21 (11)
13	B4DFL2_HUMAN	152	45,550	18 (8)	11 (7)
14	P06576_HUMAN	146	56,525	35 (10)	17 (8)
15	P09493_HUMAN	144	32,746	41 (9)	31 (7)
16	P06732_HUMAN	136	43,302	17 (6)	13 (6)
17	Q01082_HUMAN	131	275,237	16 (5)	14 (5)
18	P18206_HUMAN	131	124,292	20 (8)	20 (8)
19	P38646_HUMAN	126	73,920	24 (5)	21 (5)
20	P00505_HUMAN	114	47,886	17 (5)	13 (4)
21	A8MX12_HUMAN	114	189,126	13 (6)	12 (6)
22	Q9UJS0_HUMAN	111	74,528	9 (6)	8 (5)
23	C9JW96_HUMAN	108	26,932	9 (5)	7 (5)
24	Q13423_HUMAN	108	114,564	18 (5)	15 (5)
25	P17540_HUMAN	107	47,988	11 (3)	7 (3)
26	A2A274_HUMAN	106	88,563	27 (5)	18 (4)
27	P16219_HUMAN	99	44,611	7 (2)	4 (2)

These chaperonins were screened out using RNA immunoprecipitation and mass spectrometry.

through recruiting hnRNP-K. To verify this speculation, co-immunoprecipitation (coIP) analysis was applied. It was shown that the enriched YAP1, TAZ1, and LINC01413 in the hnRNP-K-precipitated complex were all diminished under LINC01413 depletion, and upregulating hnRNP-K partly recovered the enrichment of only YAP1/TAZ1. In contrast, ectopic LINC01413 remarkably boosted YAP1/TAZ1/LINC01413 in hnRNP-K immunoprecipitates, and sh-hnRNP-K co-transfection normalized YAP1/TAZ1 enrichment (Figure 5F). Furthermore, hnRNP-K stimulation reversed the accumulation of cytoplasmic YAP1/TAZ1 and decreased nuclear YAP1/TAZ1 caused by LINC01413 silencing, whereas hnRNP-K inhibition offset the stimulatory impact of forced LINC01413 expression on YAP1/TAZ1 nuclear translocation (Figure 5G). Such phenomena were further confirmed by western blot analyses (Figure 5H). Taken together, LINC01413 promotes YAP1/TAZ1 translocation into nucleus via an hnRNP-K-dependent manner.

The LINC01413/hnRNP-K/TAZ1/YAP1/ZEB1 Axis Stimulates Formation of a Malignant Phenotype in CRC

Subsequently, we attempted to verify whether LINC01413 functions in CRC through modulating the hnRNP-K/TAZ1/YAP1/ZEB1 axis. First, the effect of hnRNP-K on LINC01413-affected YAP1/TAZ1/ZEB1 signaling was estimated. As displayed in Figure 6A, overexpression of hnRNP-K neutralized the decrease of ZEB1 levels and the increase of p-YAP1 and p-TAZ1 levels in LINC01413-silenced LoVo cells. Accordingly, its knockdown reversed the increase of ZEB1 expression as well as the decrease of the phosphorylation levels of YAP1 and TAZ1 in HT-29 cells in the context of LINC01413 overexpression. Moreover, overexpressed hnRNP-K impaired the impact of shLINC01413 on reducing nuclear harvest of YAP1/TAZ1 and stimulating cytoplasmic accumulation of YAP1/TAZ1 in LoVo cells, whereas hnRNP-K silence manifested an opposite effect on the distribution of YAP1/TAZ1 in LINC01413-upregulated HT-29 cells (Figure 6B), revealing that the translocation of YAP1/TAZ1 is regulated by the LINC01413/hnRNP-K axis.

Thereafter, we further investigated regulation of the LINC01413/hnRNP-K axis on the biological behaviors of CRC cells. As shown in Figures 6C and CD, cell viability and apoptosis influenced by LINC01413 inhibition or overexpression were reversed in response to hnRNP-K upregulation or downregulation, respectively. Results of the Transwell assay revealed that co-transfection of pGIPZ/hnRNP-K recovered cell migration and invasion abilities of LoVo cells, which were confined by LINC01413 inhibition, and hnRNP-K silencing led to opposite results (Figures 6E and 6F). Additionally, the impact of LINC01413 silencing or enhancement on EMT process in CRC cells was counteracted by hnRNP-K overexpression or depletion, respectively (Figure 6G). Importantly, we found that expression of ZEB1 declined at both mRNA and protein levels, but levels of p-YAP1 (Ser^{127}) and p-TAZ1 (Ser^{89}) were augmented (with the mRNA and protein levels of YAP1 and TAZ1 being unaffected) in *in vivo* tumors in the context of LINC01413 knockdown (Figure S4F). In conclusion, these observations prove that LINC01413 promotes tumorigenesis and metastasis in CRC through modulating the hnRNP-K/TAZ1/YAP1/ZEB1 axis.

In summary, our study provides the first evidence that LINC01413 contributes to CRC tumorigenesis and development by recruiting hnRNP-K to promote nuclear translocation of YAP1/TAZ1 so as to inspire ZEB1 expression, thus enhancing EMT and metastasis in CRC (Figure 7).

DISCUSSION

CRC is one of the most frequent human cancers in the world and occupies fourth place in the ranking of the primary causes of cancer-associated death.[31] Upon most occasions, CRC cases are divided into four stages according to clinical symptoms, and an advanced stage usually leads to poor prognosis.[32,33] In the past decade, lncRNAs have been identified to be as important in cell biology as are miRNAs.[8] The dysregulation of lncRNAs has been suggested to be associated with a number of human diseases, cancer included.[9,34–36] For

Box 1 Sequences in LINC01413 for the Binding of hnRNP-K

Homo sapiens long intergenic non-protein coding RNA 1413 (LINC01413):

CGGTACTGAGAGCAGCCATCTTGGAGCTTGGAAACGGAGACGC
ATGTGAAGCCGTTTGCACAGTATTTTTCCCTTAAAATATGGGTG
GGGAACAAGGGGCTGTCATTTGTCTTTTTCTTTGGGATGGTGAGC
AGTGAGCAGGGCACGCCGCGAGGAGCAAACTGCAGCCCAAGC
ACACGCGGTCAGCACGCGGAGCACAGGTGGGGAGAAGCGGCT
GACGGTGGCGTGGCCCCGCGTACCTGGGTGTGGACGCCCCGCC
GCCCCGCAGGGGAAAGCTCCTGGAGTCTGGGGTCTGCTGCCCC
GAGCTCTGGGTGTCCAGCCTCTGCCTCAACTGAAATCTGAAGA
TCAGGCCAATTTCGTGGTCTCTCAGACGTCGGTAAACAAGAGGC
CTTGGCTCCTCAGGAGACCGAGAGTCTCTCACTGTACTTCCTTC
TCTTGGCTCCAAGGACATAGAACGGTTGCTATGGGGATTCCTT
CATTTGTAAAGACAATCATGCTGGCTGTTAGTGAAGAATGGCC
TGGAGCACTGGGTCTCCGCCCCAGCTGCCTGGGAGAAACTGT
GTTGAAGGAGCCATACCCCCTGGCCTCCCCTCCAGACTCCAGA
CAACCCCAGTCAGCTGGCTGGGAGGGAGGCAGAAGAGAAACA
GGGGTGCACCTGGGGCTCCTGCAGGGGTCCAGCCAGGAGATG
GGGGCAACTTGGACAGGGACGATCCTGGGAAGGGCAGGAGG
AGGAGTGAGACCTGGCCTGCCCTCTCCCTAGAGGCTCATCAGC
TCCCTGGAGACAGACACGCCCTGGTTCCTTTCCCTATGCCTGG
AGCCTGGGTACCAGCTATTGAAAACTGCTTTTCTCCTTGTTCAT
TCAAACATATTCACACATAAAGGTTGTCATTCCTTTTTGTCACT
ATTTGACAAAAATTGGATCATACCGTATACAATTCTCTGCTATT
TGTTTTGCTCACCTAACAATAAATACACTGTGAAAAATA

RPISeq predicted that hnRNP-K might bind with LINC01413 at 0–198 bp upstream of the 3′ end of LINC01413 (shown in red), with the sequence of 67–98 bp upstream of the 3′ end of LINC01413 (highlighted in yellow) maintaining the highest conservatism and the highest binding potential.

example, the lncRNA UICLM accelerates CRC liver metastasis by functioning as a competing endogenous RNA (ceRNA) of ZEB2 via sponging miR-215.[34] In the present study, we reveal a novel lncRNA LINC01413 as a facilitator in human CRC based on its promoting role in CRC cell growth and metastasis as evidenced by vitro and *in vivo* experiments.

Tumor metastasis accounts for a large proportion of cancer-caused death, and EMT is regarded as a key step in tumor metastasis because it increases cancer cell motility and invasiveness.[14] EMT occurs when epithelial cells differentiate into a mesenchymal phenotype and obtain the abilities of motility and invasion, which are strongly related to metastasis.[15] Also, EMT is controlled by a number of factors, among which ZEB1 is a primary factor of the network of transcription factors that activates EMT.[37] Additionally, lncRNAs have also been found to play roles in this process.[38] For instance, lncRNA NKILA suppresses transforming growth factor β (TGF-β)-induced EMT by blocking nuclear factor κB (NF-κB) signaling in breast cancer.[39] In our study, we found that LINC01413 also functions as a positive regulator of EMT in CRC through a ZEB1-mediated way. Also, we show that the impact of LINC01413 on EMT in CRC is closely related to the Hippo pathway, a well-known tumor suppressive pathway.[40] Furthermore, we prove that ZEB1 is transcriptionally regulated by YAP/TAZ, which is concordant with the evidence that ZEB1 is a target of YAP/TAZ, as supported by a previous report.[30]

hnRNP-K, belonging to the heterogeneous nuclear ribonucleoproteins (hnRNPs) family, is a kind of RNA-binding protein (RBP) that is of great importance in post-transcriptional events, in which lncRNAs are usually implicated.[41] In addition, aberrant hnRNP-K expression usually leads to tumorigenesis.[42–45] For example, hnRNP-K is a haplo-insufficient tumor suppressor that regulates proliferation and differentiation programs in hematologic malignancies.[46] Furthermore, hnRNP-K has been reported to function in various cellular processes, such as transcription, mRNA splicing to translation, as well as chromatin remodeling,[47] and such a role of hnRNP-K has been shown to be associated with its diverse interaction with RNA, DNA, and proteins.[48] Currently, we observed the interactivity of hnRNP-K not only with LINC01413, but also with the YAP1/TAZ1 complex, which is the key protein in Hippo signaling, revealing the cross-talk among LINC01413, hnRNP-K, YAP1, and TAZ1 in CRC cells. Recently, studies have revealed that lncRNAs can obstruct YAP phosphorylation by interacting with YAP protein through direct or indirect manners. For instance, lncARSR directly interacts with YAP to prevent LATS1-induced YAP phosphorylation.[49] lncRNA B4GALT1-AS1 enhances YAP transcriptional activity in colon cancer by interacting with YAP and inducing its nuclear translocation.[50] Also, Lin et al.[51] reported that a UCA1/AMOT/YAP interaction complex contributes to YAP dephosphorylation and nuclear translocation. Herein, we discovered that the LINC01413/hnRNP-K/YAP1/TAZ1 complex hinders YAP1 and TAZ1 phosphorylation to block their degradation, resulting in enhanced nuclear translocation of YAP1 and TAZ1 in CRC cells. On this basis, LINC01413 boosts ZEB1 transcription in CRC cells. Herein, we found that the accelerating role of LINC01413 in CRC cell growth and metastasis is mediated by ZEB1 through LINC01413/hnRNP-K/YAP1/TAZ1 signaling.

All in all, the current study primarily elaborates that the LINC01413/hnRNP-K/YAP1/TAZ1/ZEB1 axis promotes CRC progression, especially EMT-accelerated metastasis. Our findings identify LINC01413 as a novel potential prognostic and therapeutic target in CRC, exploiting a new path for the treatment of CRC patients, although there is still a long way to go until its eventual application.

MATERIALS AND METHODS

Microarray Analysis

For lncRNA analysis, three CRC tissues and corresponding non-tumor tissues were used for the microarray detection of lncRNAs. These patients had neither other underlying diseases, such as diabetes or hypertension, nor the reception of any other treatments prior to surgery. lncRNA microarray detection (H1602063, Arraystar_-Human_LncRNA_8x60k v3.0 1-color) was carried out and analyzed by Shanghai Bohao Biotechnology (Shanghai, China), and a total of 473 lncRNAs were found to be differentially expressed between tumor and non-tumor tissues, with 43 being changed 14-fold or more, among which 26 were upregulated and 17 were downregulated.

For mRNA analysis, extracted RNA was transcribed and amplified into fluorescent cRNA after removing the rRNA (mRNA-ONLY eukaryotic mRNA isolation kit, Epicenter Biotechnologies, Madison,

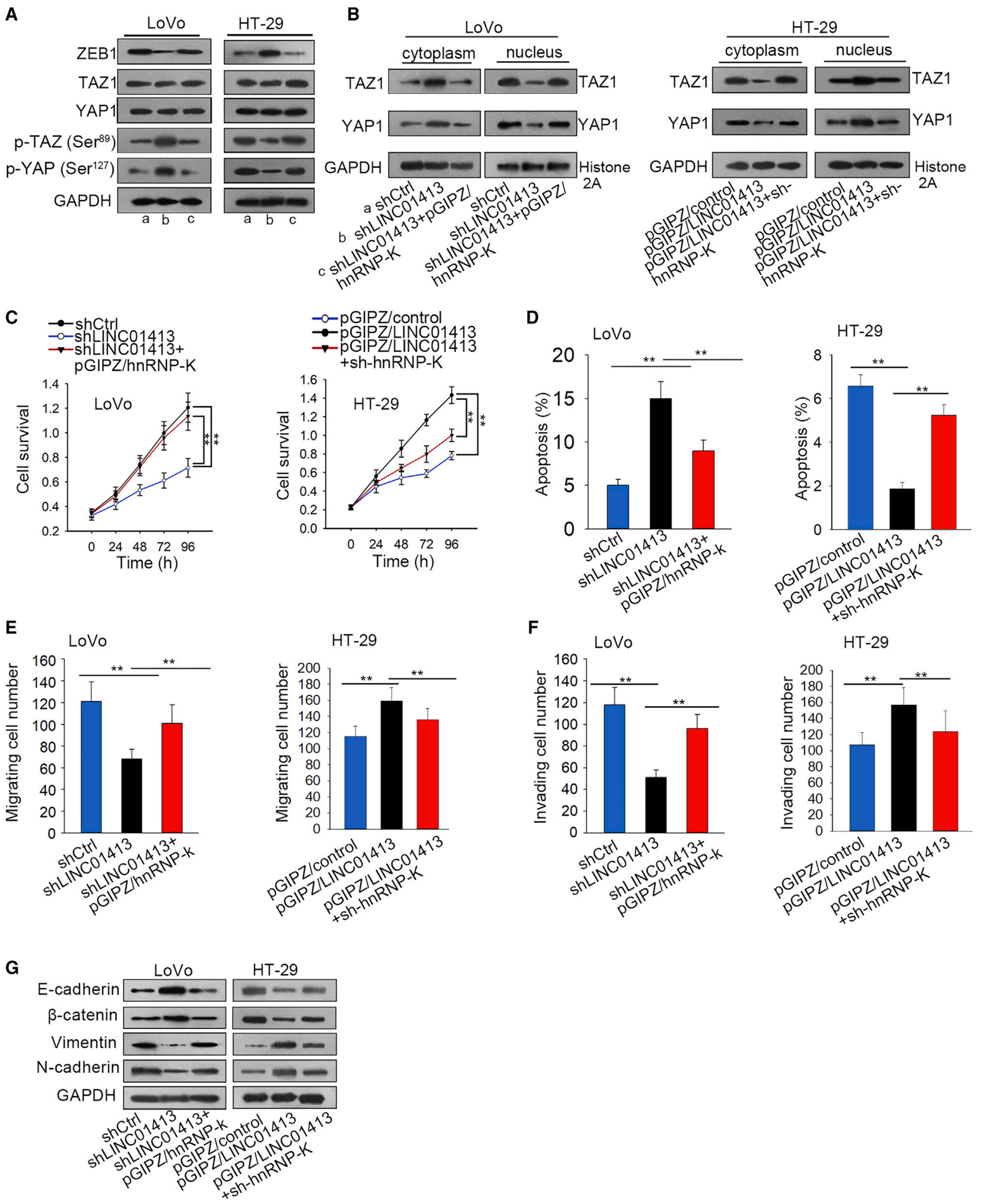

(legend on next page)

WI, USA). Then, cRNAs were tagged and hybridized onto the Human MRNA Array v3.0 (8 × 60 K, Arraystar, Rockville, MD, USA). The arrays were scanned by the Agilent G2505C scanner after washing, and the array images acquired were analyzed by the Agilent Feature Extraction software (version 11.0.1.1). The GeneSpring GX v11.5.1 software package (Agilent Technologies) was used to perform quantile normalization following data processing. Based on fold change filtering, genes with a fold change $\geq$2.0 or $\leq$0.5 were identified to be differentially expressed, and the expression profiles were visualized by using the Molecular Signatures Database (MSigDB, http://www.broadinstitute.org/msigdb). The microarray detection of mRNAs was also performed by Shanghai Bohao Biotechnology (Shanghai, China).

GO Analysis and Pathway Analysis

GO and pathway analyses were conducted as described[52] to better understand the roles of these differentially expressed genes. In this process, three independent categories, including biological process (BP), cellular component (CC), and molecular function (MF), were all derived from the GO Consortium website (http://www.geneontology.org).[52] The Database for Annotation, Visualization and Integrated Discovery (DAVID, http://david.abcc.ncifcrf.gov/) was applied to assess the changes of the upregulated and downregulated genes. The biological pathways with obvious enriched genes that were differentially expressed were analyzed by the Kyoto Encyclopedia of Genes and Genomes (KEGG) database (http://www.genome.jp/kegg/).

Tissue Specimens

A total of 92 pairs of tumor and adjacent non-tumor tissues were used in this study. These tissue specimens were obtained from CRC patients who underwent surgical operations in The First Hospital of Wenzhou Medical University. None of the patients suffered from chemotherapy or radiotherapy prior to surgery. This study was approved by the Ethics Committee of The First Hospital of Wenzhou Medical University (with the ethical approval no. YS2019-373). All patients signed the personal informed consent.

Cell Culture

The normal human colon epithelial cell line (NCM460) and five CRC cell lines (SW480, SW620, HCT-116, HT-29, and LoVo) were obtained from the American Type Culture Collection (Manassas, VA, USA). All cells were cultured in Dulbecco's modified Eagle's medium (DMEM; Invitrogen, Carlsbad, CA, USA) containing 10% fetal bovine serum (FBS; Wisent, Ottawa, ON, Canada), 100 U/mL penicillin, and 100 mg/mL streptomycin (Invitrogen), and the cells were kept under humid condition at 37°C with 5% CO_2.

Cell Transfection

Cells were inoculated in six-well plates at a concentration of 2×10^5 per well and maintained at 37°C in an incubator overnight. The silencing or overexpressing recombinant plasmids (shLINC01413 and shCtrl; pGIPZ/LINC01413 and pGIPZ/control; sh-hnRNP-K and shCtrl; pGIPZ/hnRNP-K and pGIPZ/control) were all constructed via pSUPER-EGFP1 vector. Thereafter, the plasmids were transfected into indicated cells as required by using Lipofectamine 2000 (Invitrogen), and the stably transfected ones were selected using G418 (Clontech, CA, USA). These stable cells were collected and kept for future use.

RNA Isolation and Quantitative Real-Time PCR

TRIzol reagent (Invitrogen) was used to extract total RNA from tissues and cells. NanoDrop 2000c (Thermo Scientific, Waltham, MA, USA) was applied to determine the RNA concentration and quality. After that, a reverse transcription kit (Takara, Tokyo, Japan) was used to transcribe the RNA reversely into cDNA, and quantitative real-time PCR was evaluated using SYBR Green (Takara). GAPDH was used as the normalized control. The primers used for quantitative real-time PCR are as follows:

LINC01413

Forward: 5′-AGG CAC CTA GCC TAA CAG GA-3′

Reverse: 5′-GGG CTT CAA CTC CTG ACA CA-3′

LINC02043

Forward: 5′-GAG GAA CGG AAG AAC GGA GG-3′

Reverse: 5′-GGG CTG GAA CCA GCT AAT CA-3′

LINC01836

Forward: 5′-AAC ATG GGC ACC TGG GTA TG-3′

Reverse: 5′-TAG AAT CCG TTG GGC CAC TG-3′

MIR4713

Forward: 5′-GGG CTC TAA CCT CAG GTG TT-3′

Reverse: 5′-ACC CCA CAA TTG CAG TGA CA-3′

LRP1-AS

Forward: 5′-GGG TGA CAG CCA GAC ATT CA-3′

Reverse: 5′-TGC CAT ACA GTG GGC TTC TG-3′

Figure 6. LINC01413/hnRNP-K/TAZ1/YAP1/ZEB1 Axis Stimulates the Formation of a Malignant Phenotype in CRC

(A and B) Western blot analysis reveals that LINC01413 affects the expression of ZEB1, YAP1, and TAZ1 (A) as well as the distribution of YAP1 and TAZ1 (B) via an hnRNP-K-dependent pathway. (C-D) MTT assay and flow cytometry analysis showed the cell viability and apoptosis in LINC01413-downregulated -upregulated cells were recovered after overexpression or knockdown of hnRNP-k. (E and F) The migration (E) and invasion (F) capacities of cells under different conditions were evaluated by a Transwell assay. (G) Western blot analysis determines the levels of proteins involved in EMT in CRC cells facing diverse situations. Error bars show the mean ± SD of more than three independent experiments. **p < 0.01 versus control group.

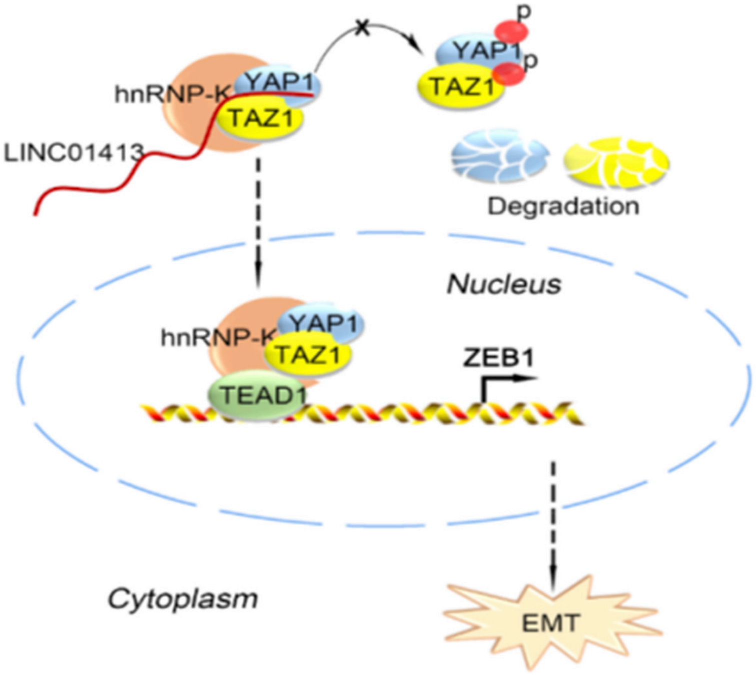

Figure 7. LINC01413 Promotes Nuclear Translocation of YAP1/TAZ1 by Recruiting hnRNP-K and Then Stimulates ZEB1 Expression, Thus Enhancing EMT and Metastasis in CRC

ZEB1

Forward: 5′-AAG TGG CGG TAG ATG GTA ATG T-3′

Reverse: 5′-AAG GAA GAC TGA TGG CTG AAA T-3′

YAP1

Forward: 5′-TAG CCC TGC GTA GCC AGT TA-3′

Reverse: 5′-TCA TGC TTA GTC CAC TGT CTGT-3′

TAZ1

Forward: 5′-ACT AAT GGT TGA TTA TCA AGA TCT TCA AGT TGG G-3′

Reverse: 5′-ACT ATT AAA TTG GAA AAA ATG TAA AAT ACT GAT GAT CAC GG-3′

GAPDH

Forward: 5′-GGA AAA GGA CAT TTC CAC CGC-3′

Reverse: 5′-AGG AGT GGG AGC ACA GGT AA-3′

MTT Assay

An MTT assay was used to determine cell viability. All of the cells were added into 96-well plates at a concentration of 5×10^3 cells/well. After a 48-h incubation, 20 μL of MTT (5 mg/mL; Sigma-Aldrich) was added into each well and the cells were then maintained in a humidified incubator. Four hours later, the supernatant was removed and 50 μL of DMSO was added to every well to dissolve the formazan. Then, the cell viability of different treated cells was determined by detecting the absorbance in the wave of 490 nm normalized to non-treated cells.

Cell Apoptosis Analysis

First, cells were placed in Petri dishes (10-cm diameter, 3×10^4 cells/dish), and cells in each dish were incubated with 2 μg/mL cisplatin for 48 h after growing to cover 80% of the entire dish. Then, flow cytometry analysis was carried out to evaluate the apoptosis rate of the cells collected from each dish using the annexin V-fluorescein isothiocyanate (FITC)/propidium iodide (PI) staining method (Beyotime, China).

Transwell Assays

For cell migration assays, cells (1×10^5) maintained in the medium without FBS were placed into the upper compartment (Millipore, Billerica, MA, USA), and the medium in the lower compartment was supplemented with 10% FBS, which functions as a chemoattractant. After incubation for 24 h, cells traveling through the pores were fixed and stained with crystal violet. Then, the cells in five random fields were counted with an inverted microscope. For cell invasion assays, all of the steps were the same as for the migration assays except that cells were seeded in the upper Transwell inserts, which had been coated with Matrigel (BD Biosciences, New York, NY, USA) for 30 min at 37°C. All data were obtained from three independent assays.

IF Staining

Cells were placed on coverslips in 24-well plates and then washed with cold PBS three times. Then, the coverslips were fixed with 4% formaldehyde and immunostained using monoclonal antibodies of E-cadherin and Vimentin. After that, DNA of these cells was stained with 4′,6-diamidino-2-phenylindole (DAPI). The Alexa Fluor 546 (red) goat anti-rabbit antibody (Molecular Probes, OR, USA) was used for E-cadherin (no. 14472, 1:50; Cell Signaling Technology, Boston, MA, USA) and Vimentin (sc-66002, 1:50; Santa Cruz Biotechnology, Santa Cruz, CA, USA). Immunofluorescence was determined using confocal microscopy (Leica TCS SP5).

In Vivo Assays

For *in vivo* xenograft experiments, a total of eight BALB/C-nu/nu mice with an age of 5 weeks were raised in a specific pathogen-free (SPF) environment with air conditioning. These mice, half males

and half females, were randomly divided into two groups (four mice per group), and then LoVo cells (1×10^6 cells per mouse) transfected with shLINC01413 or empty vector were inoculated in mice at the left flank, respectively. Then, the tumors grown in two groups were measured every 4 days until mice were sacrificed. After the mice were sacrificed, these tumors were excised, weighed, and photographed. In respect to *in vivo* metastasis experiments, LINC01413-silenced LoVo cells or the corresponding control cells were injected into the tail veins of mice. After 6 weeks of injection, all of the mice above were euthanized, and all of the tumors were finally paraffin embedded. All animal experiments in our study were approved by the Animal Welfare and Research Ethics Committee at The First Hospital of Wenzhou Medical University and were carried out strictly according to the *Guide for the Care and Use of Laboratory Animals*.

Subcellular Fractionation Location

The PARIS kit (Life Technologies, Carlsbad, CA, USA) was used to separate the nuclear and cytosolic fractions of cells on the basis of the manufacturer's recommendations.

RNA Pull-Down Assay

LINC01413 RNA was transcribed with T7 RNA polymerase (Roche) and then biotin labeled using biotin RNA labeling mix (Roche). The RNA pull-down assay was carried out by using a magnetic RNA-protein pull-down kit (Thermo Fisher Scientific) on the basis of the manufacturer's instructions. Biotinylated LINC01413 was hatched at 4°C with streptavidin beads overnight. After adding cell lysate, the system was incubated for another 4 h at 4°C. Then, beads were washed three times and the level of hnRNP-K protein in the eluted complex was analyzed using western blotting.

RIP Assays

RIP experiments were carried out by using a Magna RIP RNA-binding protein immunoprecipitation kit (Millipore, USA) under the manufacturer's protocols. hnRNP-K antibody for the RIP assay was purchased from Abcam (Cambridge, MA, USA).

CoIP Assay

Cells were lysed with lysis buffer (50 mmol/L Tris-HCl [pH 7.5], 150 mmol/L NaCl, 0.2% Nonidet P-40, 5% glycerol, 1% [v/v] Tween 20, protease inhibitor cocktail [Roche]). Then, the cell lysates were centrifuged at $12{,}000 \times g$ at 4°C for 10 min and incubated with hnRNP-K antibody (ab52600, Abcam) at 4°C for 3 h. After that, protein A/G Sepharose beads (Santa Cruz) were added and incubated overnight. On the next day, the Sepharose beads were washed three times using lysis buffer, and the proteins or RNAs immunoprecipitated by hnRNP-K were determined using western blot or northern blot analysis.

FISH

FISH assays were carried out as previously described.[53]

Western Blot

The proteins isolated from cells were separated using 10% SDS-PAGE and then transmitted to nitrocellulose (NC) membranes (Sigma, San Francisco, CA, USA). After that, the membranes were incubated with primary antibodies overnight, followed by incubation of horseradish peroxidase (HRP)-conjugated secondary antibodies (no. ab6728, 1:5,000; Abcam, UK) for another 1 h. The primary antibodies used in this study are as follows: anti-E-cadherin (no. 14472, 1:1,000), anti-β-catenin (no. 8480, 1:1,000), anti-N-cadherin (no. 13116, 1:1,000), anti-hnRNP-K (no. 9081, 1:1,000), anti-YAP (no. 14074, 1:1,000), anti-phospho-YAP (Ser^{127}) (no. 13008, 1:1,000), anti-TAZ (no. 70148, 1:1,000), anti-phospho-TAZ (Ser^{89}) (no. 59971, 1:1,000) (all of the above were purchased from Cell Signaling Technology, Boston, MA, USA); anti-Vimentin (sc-66002, 1:500; Santa Cruz Biotechnology, Santa Cruz, CA, USA); anti-ZEB1 (A301-921A, 1:2,000; Bethyl Laboratories, USA); and anti-GAPDH (G5262, 1:1,000; Sigma-Aldrich). GAPDH served as a normalized control, and proteins were captured using SuperSignal chemiluminescence substrate (Pierce, Thermo Scientific, Waltham, MA, USA) and analyzed by ImageJ (National Institutes of Health, USA).

Luciferase Reporter Assay

For luciferase reporter experiments, sequences of the ZEB1 promoter were constructed into a pGL3 vector (Promega, Madison, WI, USA). Then, pcDNA3.1, pcDNA3.1/YAP1, or pcDNA3.1/TAZ1 was respectively transfected into the HEK293T cells together with pGL3-ZEB1 promoter, using Lipofectamine 2000 (Invitrogen). 48 h after transfection, the luciferase activity was evaluated by the use of an AutoLumat LB953 luminometer (Berthold).

Statistical Analysis

All data were analyzed by using GraphPad Prism 6 or SPSS 20 (IBM, USA). Differences among groups were determined using a Student's t test, the Wilcoxon signed-rank test, Pearson's chi-square test, Pearson correlation analysis, or the nonparametric Mann-Whitney U test, as required. Kaplan-Meier analysis (log-rank test) was used to evaluate the overall survival of CRC patients. The relationship among genes was detected by Spearman's correlation analysis. Statistical significance was reached when differences were expressed with p values <0.05.

SUPPLEMENTAL INFORMATION

Supplemental Information can be found online at https://doi.org/10.1016/j.omtn.2019.11.027.

AUTHOR CONTRIBUTIONS

L. Ji designed this study. L. Ji, X.L., and Z. Zhou performed experiments, interpreted results of experiments, and analyzed data. L. Ji, Z. Zheng, L. Jin, and F.J. prepared figures and drafted the manuscript. All authors approved the final version of the manuscript.

CONFLICTS OF INTEREST

The authors declare no competing interests.

ACKNOWLEDGMENTS

This research is supported by Natural Science Foundation of Zhejiang Province (LY15C070003).

REFERENCES

1. Siegel, R., Ma, J., Zou, Z., and Jemal, A. (2014). Cancer statistics, 2014. CA Cancer J. Clin. *64*, 9–29.
2. Loree, J.M., and Kopetz, S. (2017). Recent developments in the treatment of metastatic colorectal cancer. Ther. Adv. Med. Oncol. *9*, 551–564.
3. Gkolfakis, P., Tziatzios, G., Dimitriadis, G.D., and Triantafyllou, K. (2017). New endoscopes and add-on devices to improve colonoscopy performance. World J. Gastroenterol. *23*, 3784–3796.
4. Banerjee, A., Pathak, S., Subramanium, V.D., G, D., Murugesan, R., and Verma, R.S. (2017). Strategies for targeted drug delivery in treatment of colon cancer: current trends and future perspectives. Drug Discov. Today *22*, 1224–1232.
5. Gupta, G.P., and Massagué, J. (2006). Cancer metastasis: building a framework. Cell *127*, 679–695.
6. Marchese, F.P., Raimondi, I., and Huarte, M. (2017). The multidimensional mechanisms of long noncoding RNA function. Genome Biol. *18*, 206.
7. Morlando, M., and Fatica, A. (2018). Alteration of epigenetic regulation by long noncoding RNAs in cancer. Int. J. Mol. Sci. *19*, E570.
8. Noh, J.H., Kim, K.M., McClusky, W.G., Abdelmohsen, K., and Gorospe, M. (2018). Cytoplasmic functions of long noncoding RNAs. Wiley Interdiscip. Rev. RNA *9*, e1471.
9. Schmitt, A.M., and Chang, H.Y. (2016). Long noncoding RNAs in cancer pathways. Cancer Cell *29*, 452–463.
10. Mitra, R., Chen, X., Greenawalt, E.J., Maulik, U., Jiang, W., Zhao, Z., and Eischen, C.M. (2017). Decoding critical long non-coding RNA in ovarian cancer epithelial-to-mesenchymal transition. Nat. Commun. *8*, 1604.
11. Hu, H., Wang, Y., Ding, X., He, Y., Lu, Z., Wu, P., Tian, L., Yuan, H., Liu, D., Shi, G., et al. (2018). Long non-coding RNA XLOC_000647 suppresses progression of pancreatic cancer and decreases epithelial-mesenchymal transition-induced cell invasion by down-regulating NLRP3. Mol. Cancer *17*, 18.
12. Sun, J., Hu, J., Wang, G., Yang, Z., Zhao, C., Zhang, X., and Wang, J. (2018). lncRNA TUG1 promoted KIAA1199 expression via miR-600 to accelerate cell metastasis and epithelial-mesenchymal transition in colorectal cancer. J. Exp. Clin. Cancer Res. *37*, 106.
13. Kong, J., Sun, W., Li, C., Wan, L., Wang, S., Wu, Y., Xu, E., Zhang, H., and Lai, M. (2016). Long non-coding RNA LINC01133 inhibits epithelial-mesenchymal transition and metastasis in colorectal cancer by interacting with SRSF6. Cancer Lett. *380*, 476–484.
14. Tsai, J.H., and Yang, J. (2013). Epithelial-mesenchymal plasticity in carcinoma metastasis. Genes Dev. *27*, 2192–2206.
15. Yao, D., Dai, C., and Peng, S. (2011). Mechanism of the mesenchymal-epithelial transition and its relationship with metastatic tumor formation. Mol. Cancer Res. *9*, 1608–1620.
16. Scheel, C., and Weinberg, R.A. (2012). Cancer stem cells and epithelial-mesenchymal transition: concepts and molecular links. Semin. Cancer Biol. *22*, 396–403.
17. De Craene, B., and Berx, G. (2013). Regulatory networks defining EMT during cancer initiation and progression. Nat. Rev. Cancer *13*, 97–110.
18. Zhang, P., Sun, Y., and Ma, L. (2015). ZEB1: at the crossroads of epithelial-mesenchymal transition, metastasis and therapy resistance. Cell Cycle *14*, 481–487.
19. Wang, L., Yang, F., Jia, L.T., and Yang, A.G. (2017). Missing links in epithelial-mesenchymal transition: long non-coding RNAs enter the arena. Cell. Physiol. Biochem. *44*, 1665–1680.
20. Gonzalez, D.M., and Medici, D. (2014). Signaling mechanisms of the epithelial-mesenchymal transition. Sci. Signal. *7*, re8.
21. Jung, H.Y., Fattet, L., and Yang, J. (2015). Molecular pathways: linking tumor microenvironment to epithelial-mesenchymal transition in metastasis. Clin. Cancer Res. *21*, 962–968.
22. Xu, Q., Deng, F., Qin, Y., Zhao, Z., Wu, Z., Xing, Z., Ji, A., and Wang, Q.J. (2016). Long non-coding RNA regulation of epithelial-mesenchymal transition in cancer metastasis. Cell Death Dis. *7*, e2254.
23. Ding, X.M. (2014). MicroRNAs: regulators of cancer metastasis and epithelial-mesenchymal transition (EMT). Chin. J. Cancer *33*, 140–147.
24. Hou, P., Zhao, Y., Li, Z., Yao, R., Ma, M., Gao, Y., Zhao, L., Zhang, Y., Huang, B., and Lu, J. (2014). lincRNA-ROR induces epithelial-to-mesenchymal transition and contributes to breast cancer tumorigenesis and metastasis. Cell Death Dis. *5*, e1287.
25. Harvey, K.F., Zhang, X., and Thomas, D.M. (2013). The Hippo pathway and human cancer. Nat. Rev. Cancer *13*, 246–257.
26. Pan, D. (2010). The Hippo signaling pathway in development and cancer. Dev. Cell *19*, 491–505.
27. Zanconato, F., Cordenonsi, M., and Piccolo, S. (2016). YAP/TAZ at the roots of cancer. Cancer Cell *29*, 783–803.
28. Zhao, B., Lei, Q.-Y., and Guan, K.-L. (2008). The Hippo-YAP pathway: new connections between regulation of organ size and cancer. Curr. Opin. Cell Biol. *20*, 638–646.
29. Hong, A.W., Meng, Z., and Guan, K.-L. (2016). The Hippo pathway in intestinal regeneration and disease. Nat. Rev. Gastroenterol. Hepatol *13*, 324–337.
30. Yuen, H.F., McCrudden, C.M., Huang, Y.H., Tham, J.M., Zhang, X., Zeng, Q., Zhang, S.D., and Hong, W. (2013). TAZ expression as a prognostic indicator in colorectal cancer. PLoS ONE *8*, e54211.
31. Torre, L.A., Bray, F., Siegel, R.L., Ferlay, J., Lortet-Tieulent, J., and Jemal, A. (2015). Global cancer statistics, 2012. CA Cancer J. Clin. *65*, 87–108.
32. Siegel, R.L., Ward, E.M., and Jemal, A. (2012). Trends in colorectal cancer incidence rates in the United States by tumor location and stage, 1992–2008. Cancer Epidemiol. Biomarkers Prev. *21*, 411–416.
33. Aizer, A.A., Chen, M.H., McCarthy, E.P., Mendu, M.L., Koo, S., Wilhite, T.J., Graham, P.L., Choueiri, T.K., Hoffman, K.E., Martin, N.E., et al. (2013). Marital status and survival in patients with cancer. J. Clin. Oncol. *31*, 3869–3876.
34. Chen, D.L., Lu, Y.X., Zhang, J.X., Wei, X.L., Wang, F., Zeng, Z.L., Pan, Z.Z., Yuan, Y.F., Wang, F.H., Pelicano, H., et al. (2017). Long non-coding RNA UICLM promotes colorectal cancer liver metastasis by acting as a ceRNA for microRNA-215 to regulate ZEB2 expression. Theranostics *7*, 4836–4849.
35. Dasgupta, P., Kulkarni, P., Majid, S., Shahryari, V., Hashimoto, Y., Bhat, N.S., Shiina, M., Deng, G., Saini, S., Tabatabai, Z.L., et al. (2018). MicroRNA-203 inhibits long noncoding RNA HOTAIR and regulates tumorigenesis through epithelial-to-mesenchymal transition pathway in renal cell carcinoma. Mol. Cancer Ther. *17*, 1061–1069.
36. Sun, Z.Q., Chen, C., Zhou, Q.B., Liu, J.B., Yang, S.X., Li, Z., Ou, C.L., Sun, X.T., Wang, G.X., Song, J.M., et al. (2017). Long non-coding RNA LINC00959 predicts colorectal cancer patient prognosis and inhibits tumor progression. Oncotarget *8*, 97052–97060.
37. Caramel, J., Ligier, M., and Puisieux, A. (2018). Pleiotropic roles for ZEB1 in cancer. Cancer Res. *78*, 30–35.
38. Dhamija, S., and Diederichs, S. (2016). From junk to master regulators of invasion: lncRNA functions in migration, EMT and metastasis. Int. J. Cancer *139*, 269–280.
39. Wu, W., Chen, F., Cui, X., Yang, L., Chen, J., Zhao, J., Huang, D., Liu, J., Yang, L., Zeng, J., et al. (2018). lncRNA NKILA suppresses TGF-β-induced epithelial-mesenchymal transition by blocking NF-κB signaling in breast cancer. Int. J. Cancer *143*, 2213–2224.
40. Taha, Z., Janse van Rensburg, H.J., and Yang, X. (2018). The Hippo pathway: immunity and cancer. Cancers (Basel) *10*, E94.
41. Pereira, B., Billaud, M., and Almeida, R. (2017). RNA-binding proteins in cancer: old players and new actors. Trends Cancer *3*, 506–528.
42. Gallardo, M., Hornbaker, M.J., Zhang, X., Hu, P., Bueso-Ramos, C., and Post, S.M. (2016). Aberrant hnRNP K expression: all roads lead to cancer. Cell Cycle *15*, 1552–1557.
43. Aboushousha, T., Hammam, O., Helal, N., and El Dahshan, S. (2018). Impact of cyclin D1 and heterogeneous nuclear ribonucleoprotein-K (HnRNP-K) on urinary bladder carcinogenesis. Asian Pac. J. Cancer Prev. *19*, 513–519.
44. Lu, J., and Gao, F.H. (2016). Role and molecular mechanism of heterogeneous nuclear ribonucleoprotein K in tumor development and progression. Biomed. Rep. *4*, 657–663.
45. Zhang, Z., Zhou, C., Chang, Y., Zhang, Z., Hu, Y., Zhang, F., Lu, Y., Zheng, L., Zhang, W., Li, X., and Li, X. (2016). Long non-coding RNA CASC11 interacts with hnRNP-K and activates the WNT/β-catenin pathway to promote growth and metastasis in colorectal cancer. Cancer Lett. *376*, 62–73.

46. Gallardo, M., Lee, H.J., Zhang, X., Bueso-Ramos, C., Pageon, L.R., McArthur, M., Multani, A., Nazha, A., Manshouri, T., Parker-Thornburg, J., et al. (2015). hnRNP K is a haploinsufficient tumor suppressor that regulates proliferation and differentiation programs in hematologic malignancies. Cancer Cell *28*, 486–499.

47. Bomsztyk, K., Denisenko, O., and Ostrowski, J. (2004). hnRNP K: one protein multiple processes. BioEssays *26*, 629–638.

48. Bomsztyk, K., Van Seuningen, I., Suzuki, H., Denisenko, O., and Ostrowski, J. (1997). Diverse molecular interactions of the hnRNP K protein. FEBS Lett. *403*, 113–115.

49. Qu, L., Wu, Z., Li, Y., Xu, Z., Liu, B., Liu, F., Bao, Y., Wu, D., Liu, J., Wang, A., et al. (2016). A feed-forward loop between lncARSR and YAP activity promotes expansion of renal tumour-initiating cells. Nat. Commun. *7*, 12692.

50. Zhang, Y., Fang, Z., Guo, X., Dong, H., Zhou, K., Huang, Z., and Xiao, Z. (2019). lncRNA B4GALT1-AS1 promotes colon cancer cell stemness and migration by recruiting YAP to the nucleus and enhancing YAP transcriptional activity. J. Cell. Physiol. *234*, 18524–18534.

51. Lin, X., Spindler, T.J., de Souza Fonseca, M.A., Corona, R.I., Seo, J.-H., Dezem, F.S., Li, L., Lee, J.M., Long, H.W., Sellers, T.A., et al. (2019). Super-enhancer-associated lncRNA UCA1 with AMOT to activate YAP target genes in epithelial ovarian cancer. iScience *17*, 242–255.

52. Ashburner, M., Ball, C.A., Blake, J.A., Botstein, D., Butler, H., Cherry, J.M., Davis, A.P., Dolinski, K., Dwight, S.S., Eppig, J.T., et al.; The Gene Ontology Consortium (2000). Gene ontology: tool for the unification of biology. Nat. Genet. *25*, 25–29.

53. Xiang, J.F., Yin, Q.F., Chen, T., Zhang, Y., Zhang, X.O., Wu, Z., Zhang, S., Wang, H.B., Ge, J., Lu, X., et al. (2014). Human colorectal cancer-specific CCAT1-L lncRNA regulates long-range chromatin interactions at the *MYC* locus. Cell Res. *24*, 513–531.

OMTN, Volume *19*

Supplemental Information

LINC01413/hnRNP-K/ZEB1 Axis Accelerates Cell Proliferation and EMT in Colorectal Cancer via Inducing YAP1/TAZ1 Translocation

Ling Ji, Xiang Li, Zhenhua Zhou, Zhihai Zheng, Li Jin, and Feizhao Jiang

Supplement figure 1 Heat maps of differentially expressed mRNA. The differentially expressed mRNA profiles are analyzed using high throughput chip technology. Red means highly expressed and blue means low expressed. Besides, this is the enlarged image of Fig. 4A.

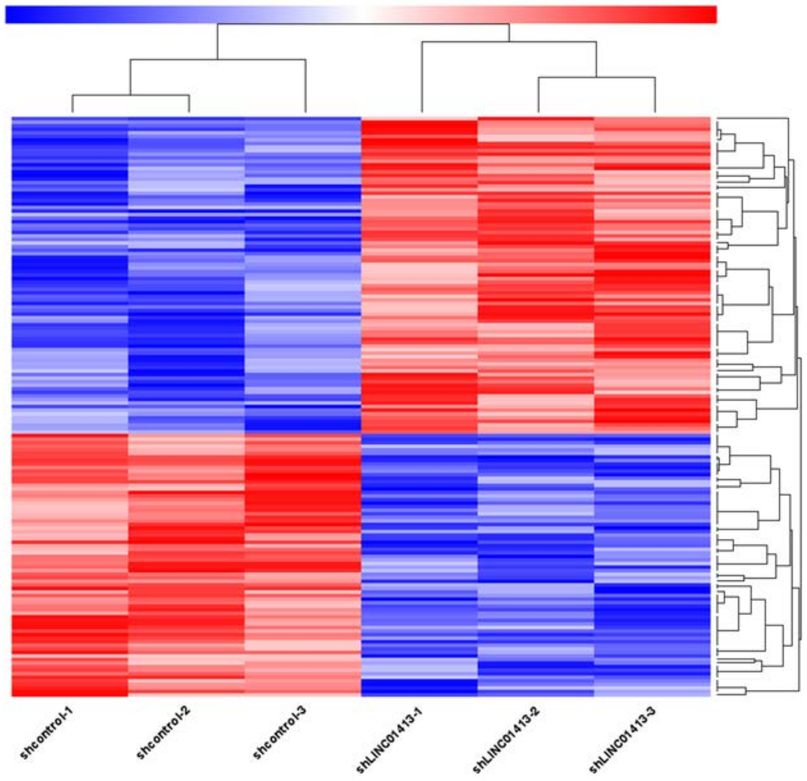

Supplement figure 2 LINC01413 mainly plays a role in Hippo pathway and cell migration process. The possible pathways or processes that could be regulated by LINC01413 which are determined by GO and pathway analysis. Besides, this is the enlarged image of Fig. 4B.

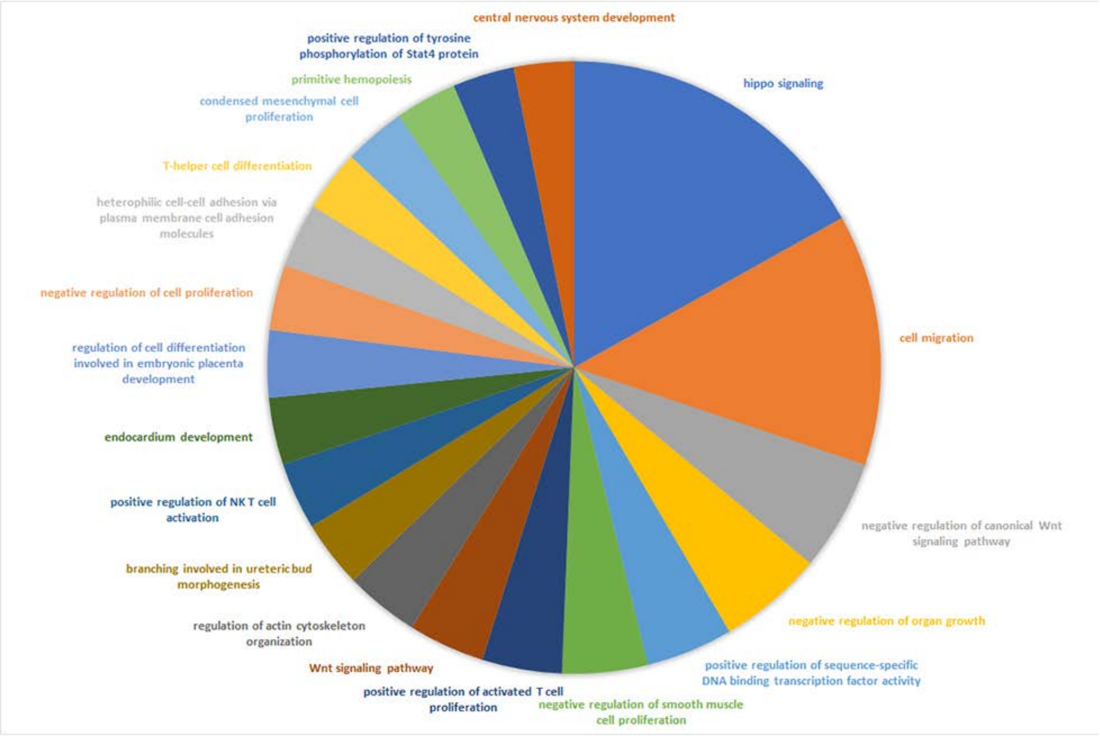

Supplement figure 3 Transcription of ZEB1 is suggested to be regulated by TEAD4. (A) UCSC predicated TEAD4 as a potential transcription factor for ZEB1. (B) The binding motif of TEAD4 obtained from JASPAR.

A

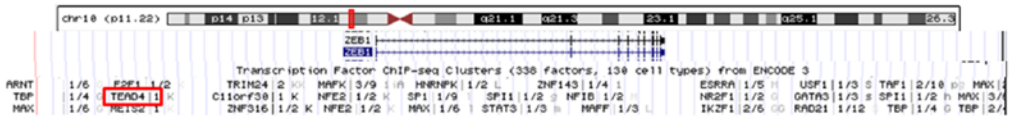

B

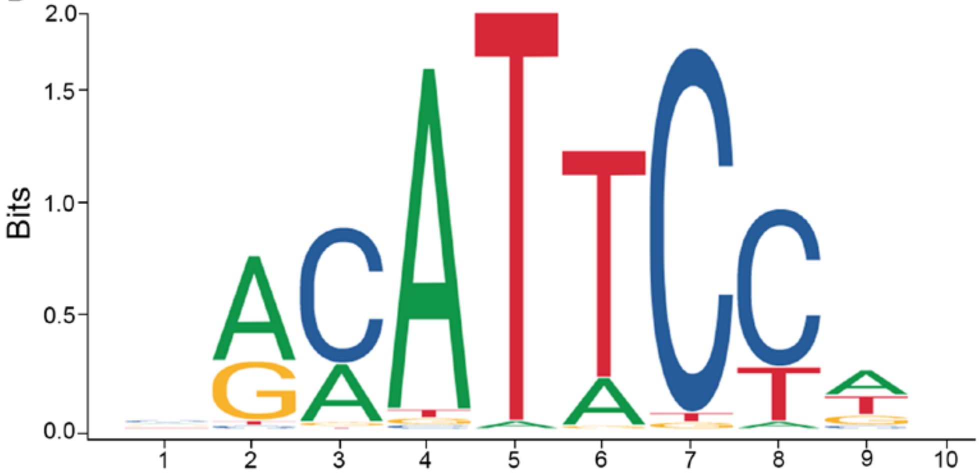

Supplement figure 4 LINC01413 regulates ZEB1 expression through hnRNP-K/YAP1/TAZ1 axis. (A-B) The impact of LINC01413 on two well-known targets of YAP/TAZ is estimated by luciferase reporter assay (A) and ChIP assay (B). (C) The result of qRT–PCR reveal that knockdown or overexpression of hnRNP-K has no influence on LINC01413 expression. (D) Western blot assay is utilized to study the effect of hnRNP-K silence on the protein levels of ZEB1, YAP1 and TAZ1 as well as the distribution of YAP1 and TAZ1 in LoVo cells. (E) Western blot results show that overexpression of hnRNP-K in HT-29 cells promotes the level of ZEB1, p-YAP1 and p-TAZ1 as well as the translocation of YAP1/TAZ1. (F) RNA expression of LINC01413, ZEB1, YAP1 and TAZ1 in tumors obtained from in vivo experiments is tested by qRT-PCR, and the protein levels of ZEB1, YAP1, TAZ1, p-YAP1 (Ser^{127}) and p-TAZ1 (Ser^{89}) in above tumors are detected through western blot. Error bars show the mean ±SD of more than three independent experiments. **P < 0.01 vs. control group.

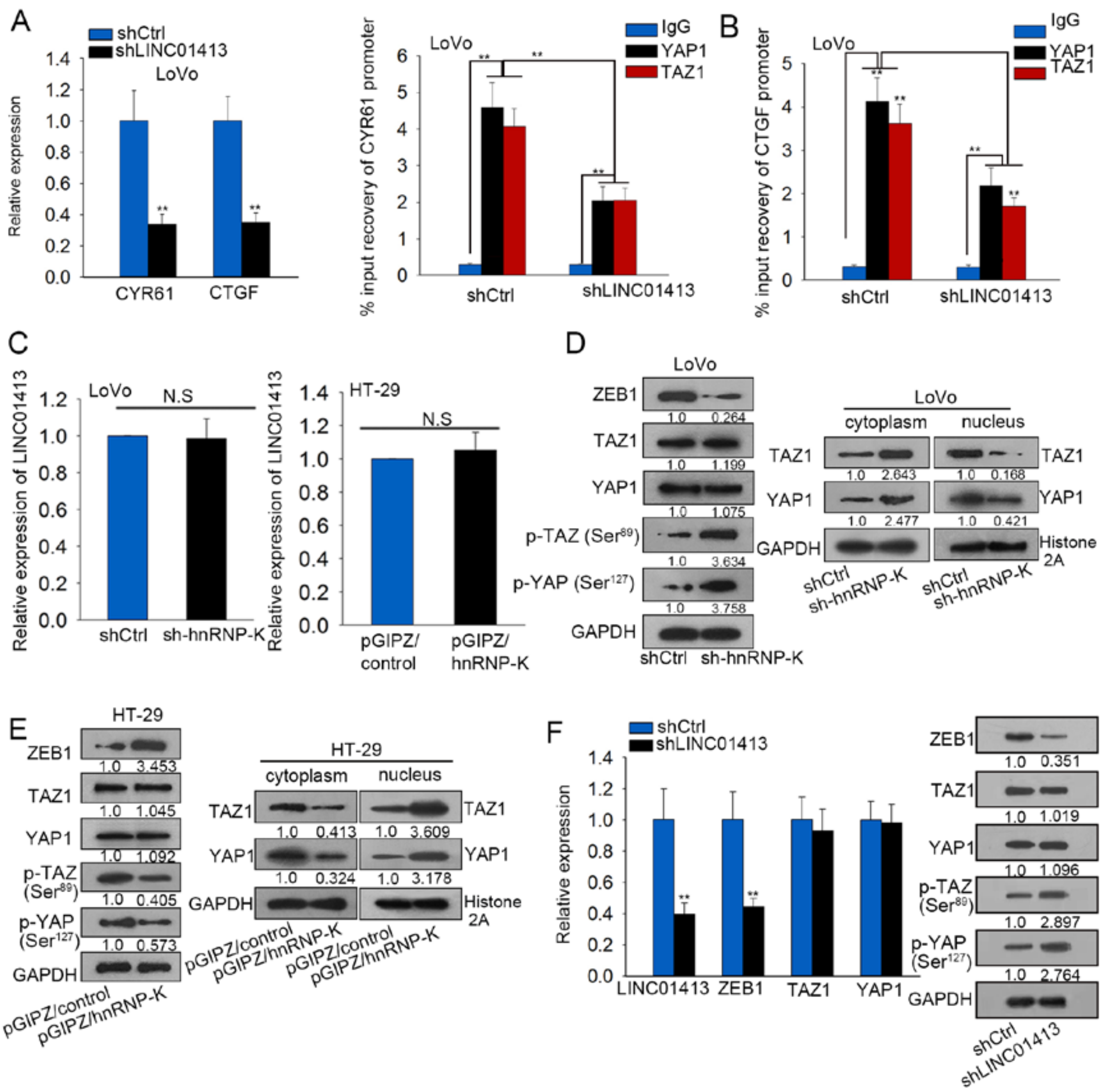

A
shCtrl
shLINC01413
LoVo
Relative expression
CYR61
CTGF
% input recovery of CYR61 promoter
IgG
YAP1
TAZ1
shCtrl
shLINC01413
B
% input recovery of CTGF promoter
C
LoVo
N.S
Relative expression of LINC01413
sh-hnRNP-K
HT-29
pGIPZ/control
pGIPZ/hnRNP-K
D
ZEB1
1.0 0.264
TAZ1
1.0 1.199
YAP1
1.0 1.075
p-TAZ (Ser89)
1.0 3.634
p-YAP (Ser127)
1.0 3.758
GAPDH
cytoplasm
nucleus
1.0 2.643
1.0 0.168
1.0 2.477
1.0 0.421
Histone 2A
E
1.0 3.453
1.0 1.045
1.0 1.092
1.0 0.405
1.0 0.573
1.0 0.413
1.0 3.609
1.0 0.324
1.0 3.178
F
LINC01413
1.0 0.351
1.0 1.019
1.0 1.096
1.0 2.897
1.0 2.764

Supplementary Figure 5 Quantification of protein bands in Figure 2L (A), Figure 3F (B), Figure 4C (C), Figure 4D (D), Figure 5H (E), Figure 6A (F), Figure 6B (G), Figure 6G (H), Figure S4D (I), Figure S4E (J), Figure S4F (K). **P < 0.01 vs. control group.

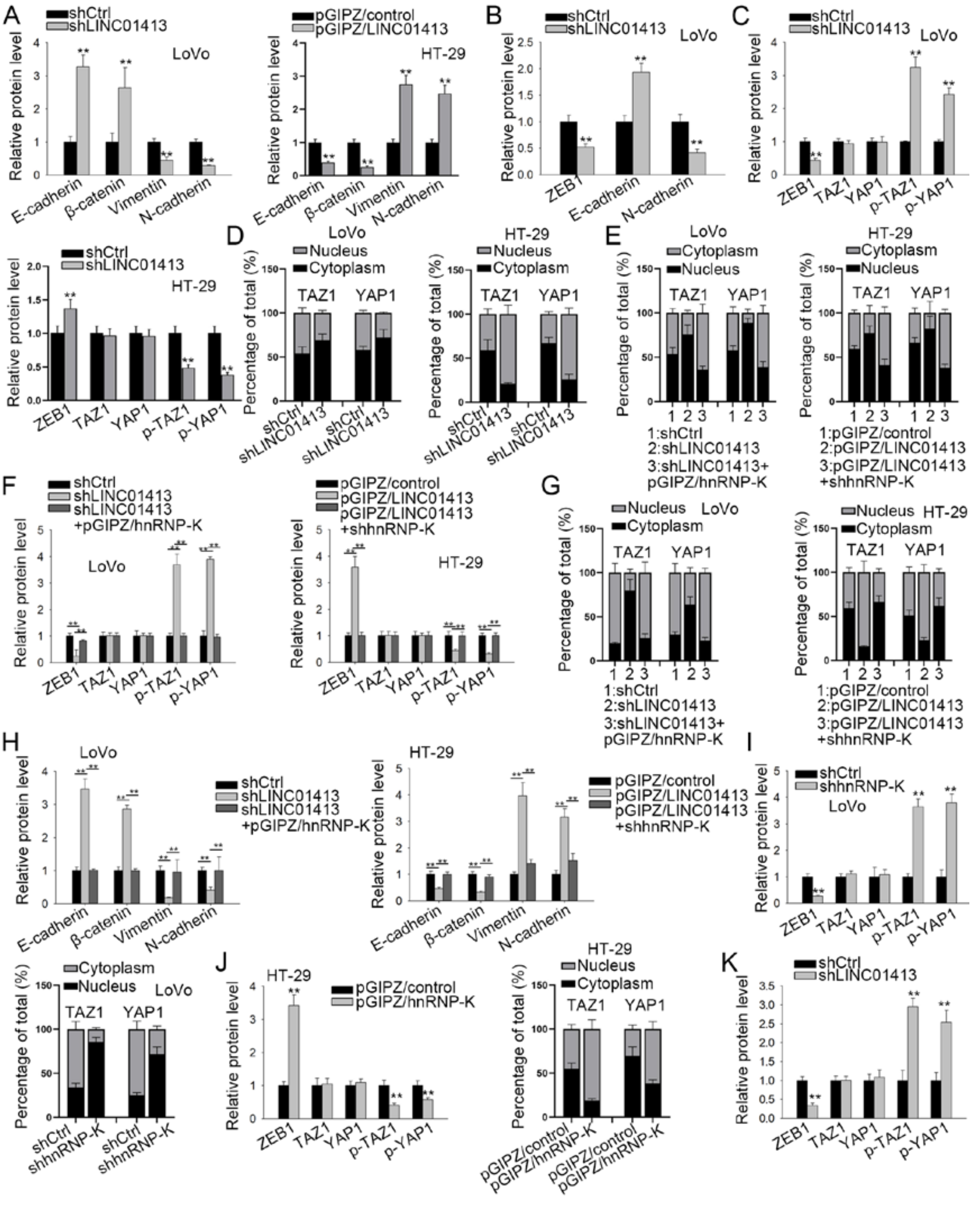

Supplement: Document S2. Article plus Supplemental Information [file mmc2.pdf]
